# C-type natriuretic peptide co-ordinates cardiac structure and function

**DOI:** 10.1093/eurheartj/ehz093

**Published:** 2019-03-21

**Authors:** Amie J Moyes, Sandy M Chu, Aisah A Aubdool, Matthew S Dukinfield, Kenneth B Margulies, Kenneth C Bedi, Kairbaan Hodivala-Dilke, Reshma S Baliga, Adrian J Hobbs

**Affiliations:** 1 William Harvey Research Institute, Barts and The London School of Medicine and Dentistry, Queen Mary University of London, Charterhouse Square, London EC1M 6BQ, UK; 2 Heart Failure and Transplant Program, Perelman School of Medicine, University of Pennsylvania, Translational Research Center, 3400 Civic Center Boulevard, Philadelphia, PA 19104, USA; 3 Barts Cancer Institute, Barts and The London School of Medicine and Dentistry, Queen Mary University of London, Charterhouse Square, London EC1M 6BQ, UK

**Keywords:** Natriuretic peptide, Natriuretic peptide receptor, Endothelium, Ischaemia/reperfusion injury, Heart failure, Cardiomyocyte

## Abstract

**Aims:**

C-type natriuretic peptide (CNP) is an essential endothelium-derived signalling species that governs vascular homoeostasis; CNP is also expressed in the heart but an intrinsic role for the peptide in cardiac function is not established. Herein, we employ unique transgenic strains with cell-specific deletion of CNP to define a central (patho)physiological capacity of CNP in maintaining heart morphology and contractility.

**Methods and results:**

Cardiac structure and function were explored in wild type (WT), cardiomyocyte (cmCNP^−/−^), endothelium (ecCNP^−/−^), and fibroblast (fbCNP^−/−^)—specific CNP knockout mice, and global natriuretic peptide receptor (NPR)-B^−/−^, and NPR-C^−/−^ animals at baseline and in experimental models of myocardial infarction and heart failure (HF). Endothelium-specific deletion of CNP resulted in impaired coronary responsiveness to endothelium-dependent- and flow-mediated-dilatation; changes mirrored in NPR-C^−/−^ mice. *Ex vivo*, global ischaemia resulted in larger infarcts and diminished functional recovery in cmCNP^−/−^ and NPR-C^−/−^, but not ecCNP^−/−^, vs. WT. The cardiac phenotype of cmCNP^−/−^, fbCNP^−/−^, and NPR-C^−/−^ (but not ecCNP^−/−^ or NPR-B^−/−^) mice was more severe in pressure overload- and sympathetic hyperactivation-induced HF compared with WT; these adverse effects were rescued by pharmacological CNP administration in WT, but not NPR-C^−/−^, mice. At a molecular level, CNP/NPR-C signalling is impaired in human HF but attenuates activation of well-validated pro-hypertrophic and pro-fibrotic pathways.

**Conclusion:**

C-type natriuretic peptide of cardiomyocyte, endothelial and fibroblast origins co-ordinates and preserves cardiac structure, function, and coronary vasoreactivity via activation of NPR-C. Targeting NPR-C may prove an innovative approach to treating HF and ischaemic cardiovascular disorders.

**Figure:**
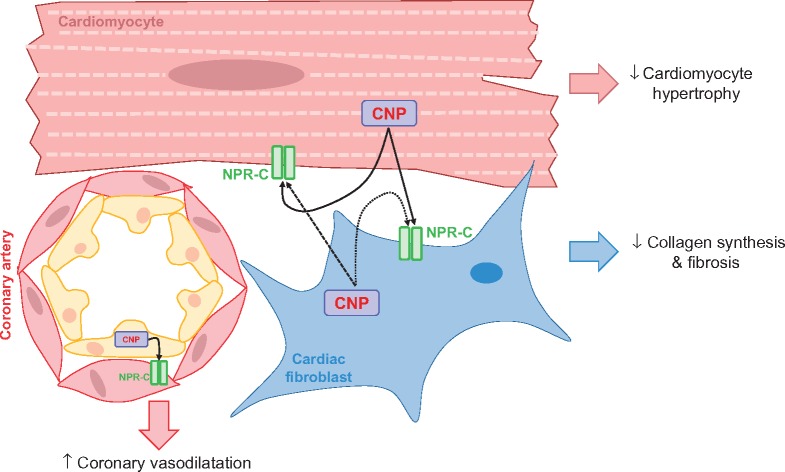

**See page 1021 for the editorial comment on this article (doi: 10.1093/eurheartj/ehz142)**

## Introduction

C-type natriuretic peptide (CNP) plays a key role in regulating vascular homoeostasis; the peptide controls local blood flow and systemic blood pressure, reduces the reactivity of leucocytes and platelets, and prevents the development of atherogenesis and aneurysm.^1–3^ The expression of CNP in endothelial cells accounts for its predominant localization in mammals (in addition to the CNS), but CNP is also found in cardiomyocytes^4^^,^^5^ and levels are up-regulated in failing hearts.^6^^,^^7^ In accord with this additional cardiac localization, CNP has an established pharmacodynamic profile in modulating heart structure and function. For example, acutely CNP primarily exerts a negative inotropic and chronotropic action, partly via inhibition of L-type calcium currents.^8^^,^^9^ In the longer term, over-expression of a dominant negative form of natriuretic peptide receptor (NPR)-B (a guanylyl cyclase-coupled cognate receptor for CNP^10^^–^^12^) in cardiomyocytes results in accelerated development of cardiac hypertrophy, fibrosis, and contractile dysfunction.^13^ Indeed, there appears to be greater expression of NPR-B vs. NPR-A (the cognate receptor for atrial and brain natriuretic peptides, ANP and BNP) during the development of cardiac hypertrophy, raising the possibility that CNP takes on the mantle of natriuretic peptide guardian of cardiac integrity.^14^ This concept is reinforced by the observations that CNP protects against myocardial infarction (MI)-induced hypertrophy^15^^,^^16^, that cardiac production of CNP increases substantially and correlates with severity in patients with heart failure (HF),^17–19^ and that the chimeric CD-NP exerts a potent beneficial effect in pre-clinical models of cardiac fibrosis.^20^ Our own work has shown that administration of synthetic CNP protects against MI via activation of NPR-C (i.e. a cyclic guanosine-3’,5’-monophosphate (cGMP)-independent action).^21^ Evidence also supports a role for CNP in the right ventricle and in the pulmonary circulation.^22–24^ Thus, there is strong evidence supporting a role for CNP in both right- and left-heart morphology and contractility. Consequently, the peptide has been tentatively termed a ‘cardiac natriuretic peptide,^25^; yet, a (patho)physiological function in this context has not been established.

**Translational perspective**

C-type natriuretic peptide (CNP) is a critical endothelium-derived signalling species governing vascular homoeostasis; however, an analogous role for the peptide in regulating heart structure and function is not established. Exploiting unique, cell-specific transgenic strains this work defines a pivotal (patho)physiological capacity of CNP to maintain cardiac morphology, ventricular contractility, and coronary microvascular reactivity. These intrinsic protective functions are mediated via natriuretic peptide receptor (NPR)-C, which is shown to be localized to cardiomyocytes and cardiac fibroblasts, and up-regulated in human failing hearts. Moreover, the study proffers pharmacological proof-of-concept that targeting NPR-C is an innovative therapeutic approach for heart failure and ischaemic cardiovascular disorders.

Herein, we employed *in vitro* and *in vivo* models and unique transgenic strains with cardiomyocyte (cmCNP^−/−^), endothelial (ecCNP^−/−^), and fibroblast (fbCNP^−/−^)—specific CNP deletion, to define the peptide as a critical player in cardiac structure, ventricular contractility, and coronary reactivity; additionally, proof-of-concept is demonstrated for pharmacological targeting of this novel pathway in HF and ischaemia cardiovascular disorders.

## Methods

### Experimental heart failure models

Pressure overload (abdominal aortic constriction; AAC) and sympathetic hyperactivation (isoprenaline; ISO) models of left ventricular hypertrophy (LVH) and cardiac dysfunction were employed as previously described^26^ (see Supplementary material online for further information).

### Primary cardiomyocyte and cardiac fibroblast isolation and culturing

The Pierce primary cardiomyocyte isolation kit (Thermo Scientific, Loughborough, UK) was used to isolate neonatal cardiomyocytes from wild type (WT) and cmCNP^−/−^ mice. Cardiac fibroblasts from adult WT and fbCNP^−/−^ animals were isolated by outgrowth from 1 mm^3^ sections of heart tissue. Further information is provided in Supplementary material online.

### Quantitative RT-PCR and immunoblotting

mRNA and protein expression were analysed using standard protocols (explicit information is provided in Supplementary material online). Specific primers for hypertrophic and fibrotic markers and housekeeping genes RLP-19 and β-actin are detailed in Supplementary material online, *Table S1*.

### *Ex vivo* assessment of coronary vascular reactivity and ischaemia/reperfusion injury

Coronary reactivity and myocardial ischaemia/reperfusion (I/R) injury were evaluated in murine hearts set-up in Langendorff mode as we have described previously.^21^^,^^26^ More detailed protocols are provided in the Supplementary material online.

### Data analysis

All data are reported as mean ± standard deviation, where *n* is the number of mice used. Statistical analyses were conducted using GraphPad Prism (version 7; GraphPad software, CA, USA). For comparison of two groups of data, a two-tailed, unpaired Student’s *t*-test was used. When comparing three or more groups of data one-way or two-way ANOVA followed by a Šídák multiple comparisons test was used with adjustment for multiplicity. *P*-value <0.05 was considered statistically significant and the *P*-values presented in each Figure indicate all comparisons undertaken.

## Results

### Genetic deletion of C-type natriuretic peptide from cardiomyocytes has modest effects on basal cardiac function

**Figure 1:**
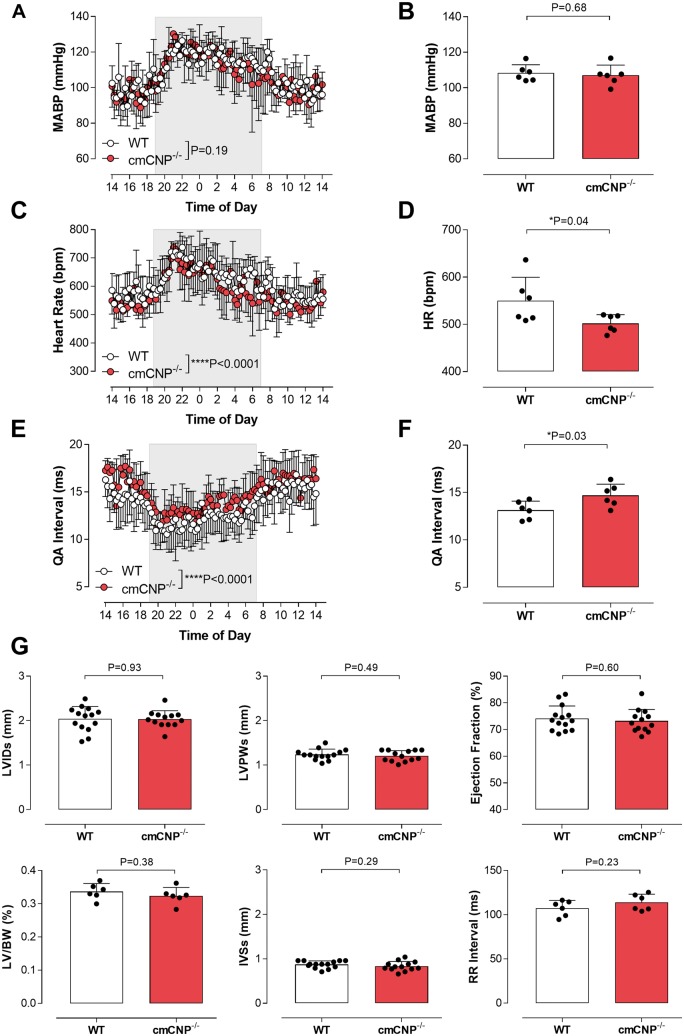
Cardiomyocyte-specific ablation of CNP has modest effects on basal cardiac function. 24 hr and mean radiotelemetry evaluation of (*A* and *B*) MABP, (*C* and *D*) heart rate and (*E* and *F*) QA interval in WT and cmCNP^−/−^ mice. (*G*) Echocardiographic analyses of left ventricular internal diameter at systole (LVIDs), left ventricular posterior wall diameter at systole (LVPWs), ejection fraction, left ventricle to body weight ratio (LV/BW), intraventricular septum diameter at systole (IVSs) and RR interval in WT and cmCNP^−/−^ animals. Data are presented as mean ± SD and analysed using two-way ANOVA with Šídák *post-hoc* test (*A*, *C* and *E*) or Student's *t*-test (*B*, *D*, *F* and *G*). Each statistical comparison undertaken has an assigned *P* value (adjusted for multiplicity).

Loss of cardiomyocyte CNP did not significantly alter any cardiac echocardiographic parameters with the exception of an increase in RR and QA interval, indicative of a basal decrease in heart rate (HR) (*Figure 1* and Supplementary material online, *Table S2*). This was substantiated by radiotelemetric analysis; cmCNP^−/−^ mice maintained a normal circadian rhythm and exhibited no difference in blood pressure (*Figure 1*) but had a significantly lower heart rate (∼20 b.p.m.) compared to WT littermates (*Figure 1*).

### Cardiomyocyte-specific deletion of C-type natriuretic peptide worsens phenotype following cardiac stress

**Figure 2:**
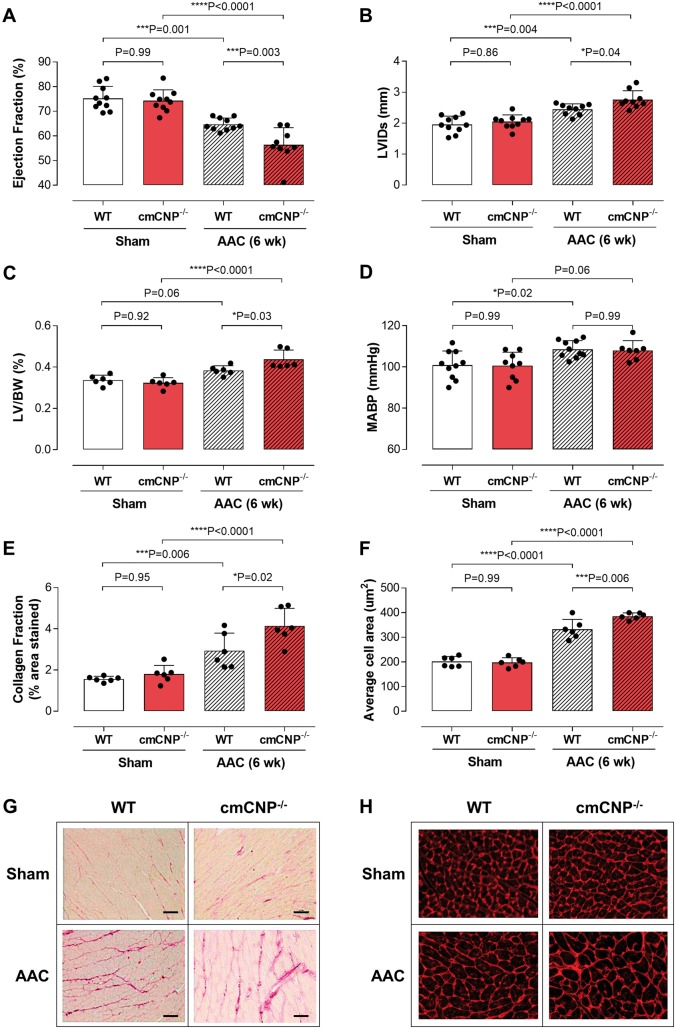
Cardiomyocyte-specific deletion of CNP worsens the cardiac response to pressure-overload. Ejection fraction (*A*), left ventricular internal diameter at systole (LVIDs; *B*), left ventricle to body weight ratio (LV/BW; *C*), mean arterial blood pressure (MABP; *D*), fibrotic burden (collagen fraction; *E* and *G*; scale bar = 50 μm) and cardiomyocyte size (*F* and *H*) in WT and cmCNP^−/−^ animals exposed to 6 weeks abdominal aortic constriction (AAC). Data are presented as mean ± SD and analysed using one-way ANOVA with Šídák *post-hoc* test. Each statistical comparison undertaken has an assigned *P* value (adjusted for multiplicity).

**Figure 3:**
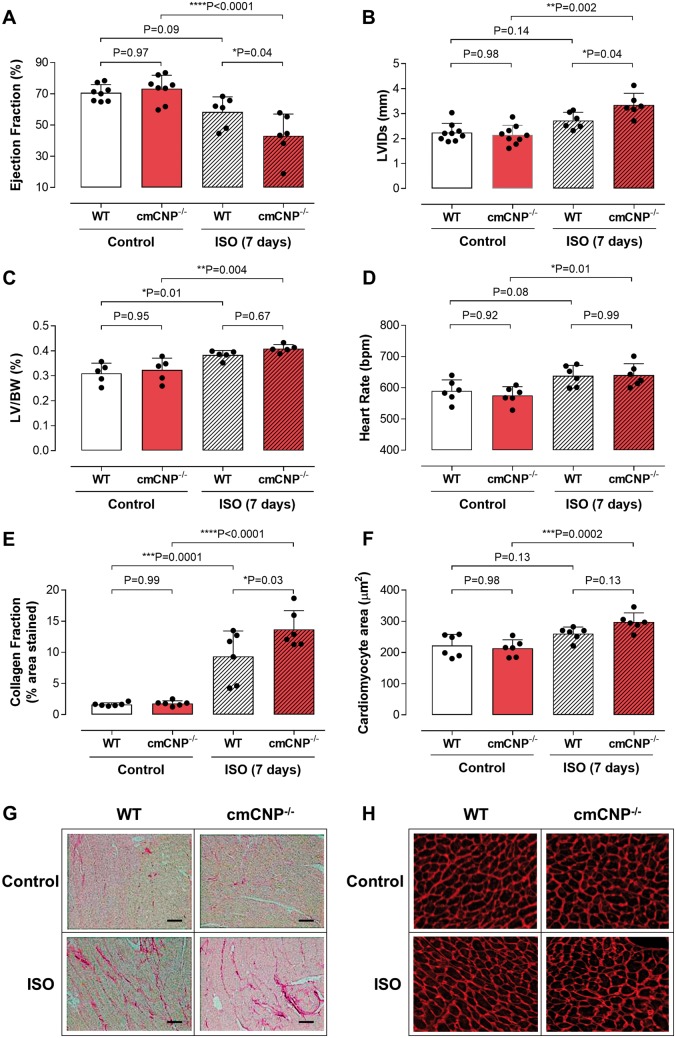
Cardiomyocyte-specific deletion of CNP worsens the cardiac response to sympathetic hyperactivation. Ejection fraction (*A*), left ventricular internal diameter at systole (LVIDs; *B*), left ventricle to body weight ratio (LV/BW; *C*), heart rate (HR; *D*), fibrotic burden (collagen fraction; *E* and *G*; scale bar = 50 μm) and cardiomyocyte size (*F* and *H*) in WT and cmCNP^−/−^ animals exposed to 7 days isoprenaline (ISO; 20mg/kg/day). Data are presented as mean ± SD and analysed using one-way ANOVA with Šídák *post-hoc* test. Each statistical comparison undertaken has an assigned *P* value (adjusted for multiplicity).

A common detrimental phenotype manifested in cmCNP^−/−^ mice in response to cardiac stress. Breeding females (heterozygous) animals exhibited a progressive deterioration in contractile function and LV dilatation with successive pregnancies, which resulted in significant mortality (Supplementary material online, *Figure S2*). This deleterious response was mirrored in two independent pre-clinical models of HF; pressure overload and sympathetic hyperactivation. Cardiomyocyte-specific deletion of CNP resulted in a significantly greater reduction in ejection fraction, exacerbated left ventricular (LV) dilatation, and more pronounced fibrosis (i.e. collagen deposition) and cardiomyocyte enlargement compared with WT littermates (*Figures 2 and *3; with a trend towards greater LV weight). The adverse outcome in cmCNP^−/−^ animals neither resulted from an indirect effect on blood pressure (pressure overload; *Figure 2*) nor changes in sympathetic responsiveness (i.e. HR; isoprenaline; *Figure 3*).

### Natriuretic peptide receptor-C activation underpins the cardioprotective function of C-type natriuretic peptide

**Figure 4:**
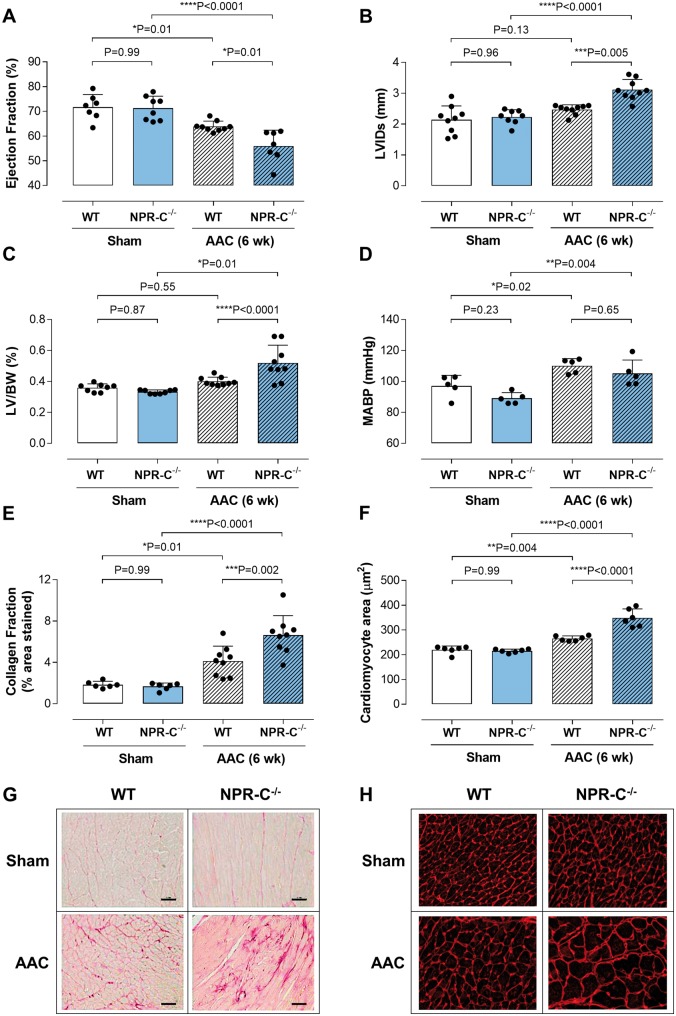
Global deletion of NPR-C worsens the cardiac response to pressure-overload. Ejection fraction (*A*), left ventricular internal diameter at systole (LVIDs; *B*), left ventricle to body weight ratio (LV/BW; *C*), mean arterial blood pressure (MABP; *D*), fibrotic burden (collagen fraction; *E* and *G*; scale bar = 50 μm) and cardiomyocyte size (*F* and *H*) in WT and NPR-C^−/−^ animals exposed to 6 weeks abdominal aortic constriction (AAC). Data are presented as mean ± SD and analysed using one-way ANOVA with Šídák *post-hoc* test. Each statistical comparison undertaken has an assigned i value (adjusted for multiplicity).

**Figure 5:**
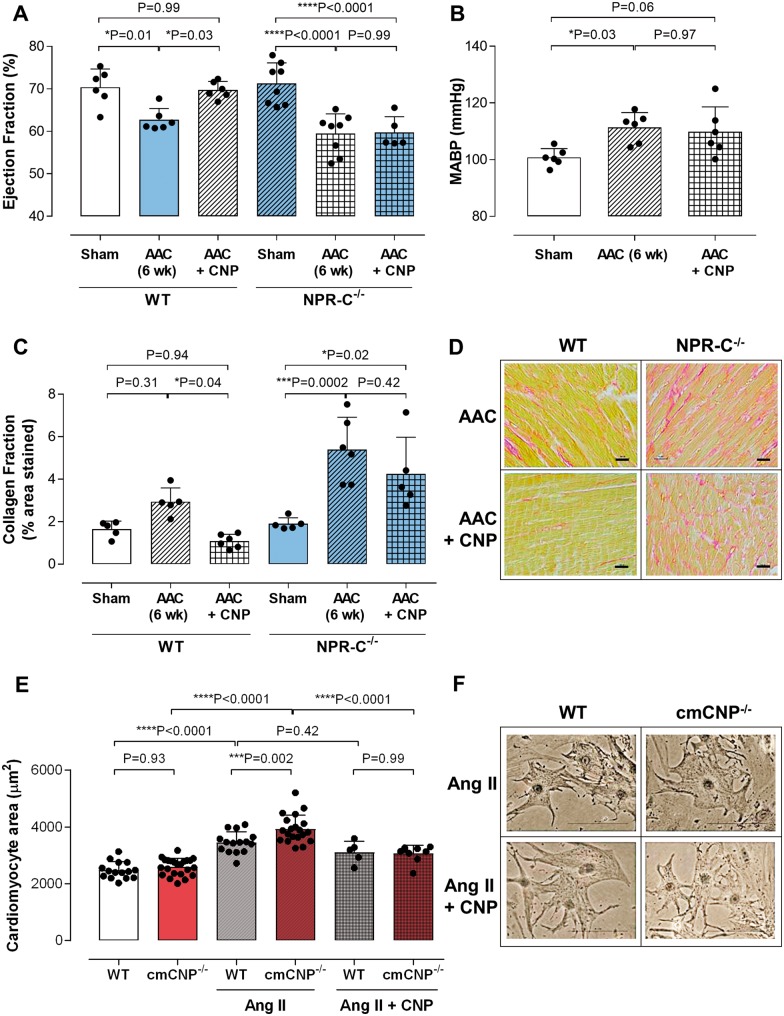
Pharmacological administration of CNP rescues the detrimental cardiac phenotype in response to pressure-overload in wild type, but not NPR-C^−/−^, mice. Ejection fraction (*A*), mean arterial blood pressure (MABP; *B*), and fibrotic burden (collagen fraction; *C* and *D*; scale bar = 50 μm) in WT or NPR-C^−/−^ animals exposed to 6 weeks abdominal aortic constriction (AAC) in the absence and presence of CNP (0.2 mg/kg/day; s.c. by osmotic minipump, initiated 3 weeks following AAC surgery and maintained throughout the study). Intrinsic hypertrophic response to Angiotensin (Ang) II and the effect of CNP (100nM) on cardiomyocytes isolated from WT and cmCNP^−/−^ mice (*E* and *F*). Data are presented as mean ± SD and analysed using one-way ANOVA with Šídák *post-hoc* test. Each statistical comparison undertaken has an assigned *P* value (adjusted for multiplicity).

Natriuretic peptide receptor-C^−/−^ mice exhibited a significantly worse phenotype in all aspects of cardiac structure and function in response to pressure overload (*Figure 4*); indeed, this was arguably more severe than that apparent in cmCNP^−/−^ animals (*Figures 2*and**3). In sharp contrast, the phenotype of NPR-B^−/−^ mice in response to sympathetic hyperactivation was mild or non-existent (Supplementary material online, *Figure S3*; the dwarfism and early death in NPR-B^−/−^^11^ severely limits study of cardiovascular biology but it was feasible to implant osmotic minipumps to deliver isoprenaline). Furthermore, therapeutic delivery of CNP (resulting in ∼10-fold increase in circulating [CNP]; Supplementary material online, *Table S3*) was able to substantially reverse the cardiac structural and functional deficits resulting from pressure overload in WT, but not NPR-C^−/−^ mice (*Figure 5*), confirming that activation of this cognate receptor is responsible for conveying the cardioprotective effects of CNP.

**Figure 6:**
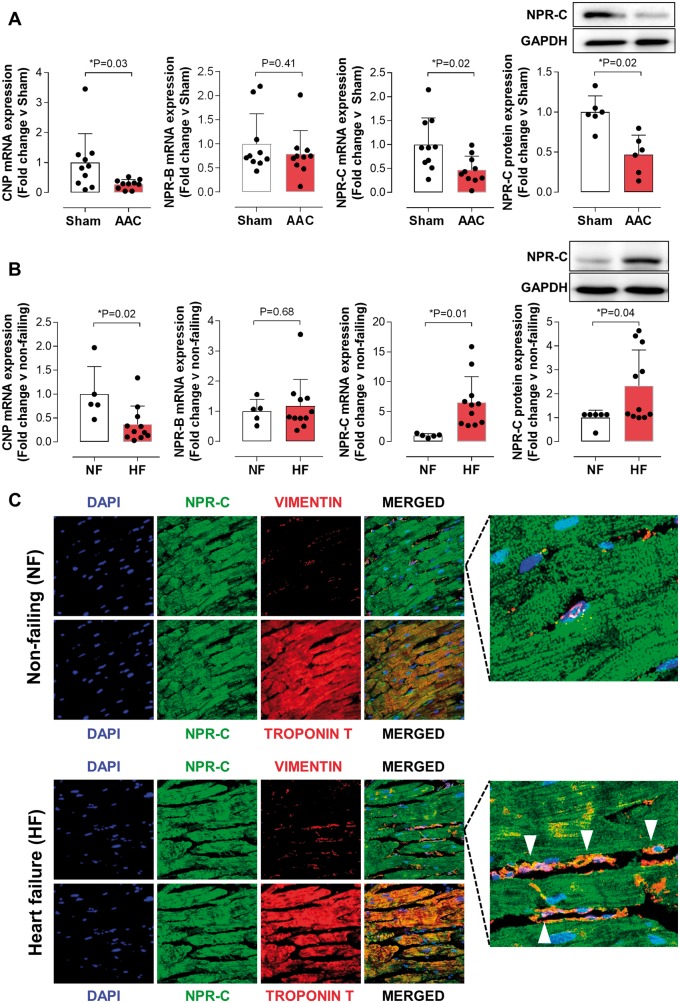
Expression and co-localization of CNP and NPR-C are altered in human heart failure. CNP, NPR-B and NPR-C mRNA (and protein) expression in murine pressure overload -induced (6 weeks abdominal aortic constriction, AAC) heart failure (*A*) and in human non-failing (NF) and failing (HF) hearts (*B*). NPR-C is highly expressed on cardiomyocytes in both non-failing and failing hearts but co-localizes with cardiac fibroblasts in heart failure patients (*C*; cardiomyocyte marker troponin T; fibroblast marker vimentin; scale bars, 50x magnification; white triangles highlight NPR-C co-localization in fibroblasts in HF). Data are presented as mean ± SD and analysed using Student's *t*-test.

In human hearts the primary cellular localization of NPR-C is the cardiomyocyte, with greater expression in failing vs. healthy hearts (albeit reduced in murine models). Interestingly, however, cardiac fibroblast co-localization of NPR-C appears to be exclusive to disease (*Figure 6*). Indeed, CNP expression is reduced in failing hearts in both humans and mouse models, whereas NPR-B levels remain consistent across species and pathological status (*Figure 6*). Despite this profile, circulating CNP concentrations were unchanged in WT, cmCNP^−/−^, or NPR-C^−/−^ mice following AAC (Supplementary material online, *Figure S4*). Moreover, plasma levels of ANP and BNP were consistent across genotypes following AAC, although there was some evidence of a subtle up-regulation of both peptides in response to loss of cardiomyocyte-derived CNP (Supplementary material online, *Figure S4*).

### C-type natriuretic peptide prevents hypertrophy in isolated cardiomyocytes

Basal cardiomyocyte size was not different between WT and cmCNP^−/−^, but the *in vitro* hypertrophic response to Angiotensin (Ang) II was significantly greater in cmCNP^−/−^ (*Figure 5*). Moreover, the hypertrophic response to Ang II in cmCNP^−/−^ cells could be rescued to WT levels with the addition of exogenous CNP (*Figure 5*), confirming a key anti-hypertrophic activity of the peptide.

### The beneficial effects of cardiomyocyte-derived C-type natriuretic peptide are not NO-dependent

Since G_i_-coupled receptors (including NPR-C) have been shown to signal via endothelial nitric oxide synthase (eNOS) phosphorylation, the salutary effect of exogenous CNP in pressure overload-induced HF was examined in the presence of NOS inhibition. In this setting, the protective capacity of CNP was maintained (Supplementary material online, *Figure S5*), intimating this beneficial pharmacodynamic action is not dependent on secondary generation of NO.

### The cardioprotective effects of C-type natriuretic peptide are linked to established hypertrophic and/or fibrotic pathways

The favourable actions of CNP were confirmed at a more molecular level by comparing the expression of pro-hypertrophic and pro-fibrotic markers/drivers in cmCNP^−/−^, fbCNP^−/−^, and NPR-C^−/−^ animals. In all genotypes, ANP (↑), Col1α1 (↑), SERCA-2 (↓), and βMHC (↑) were altered in an analogous fashion; each of which is known to be modified in human HF and contribute to ventricular dysfunction (Supplementary material online, *Figure S6*). Intriguingly, the effects on pro-fibrotic mediators were split; thus, fibronectin expression was increased in cmCNP^−/−^ and NPR-C^−/−^ mice, whereas TGFβ was up-regulated in fbCNP^−/−^ and NPR-C^−/−^ animals.

### A complementary role for fibroblast-derived C-type natriuretic peptide in heart failure

**Figure 7:**
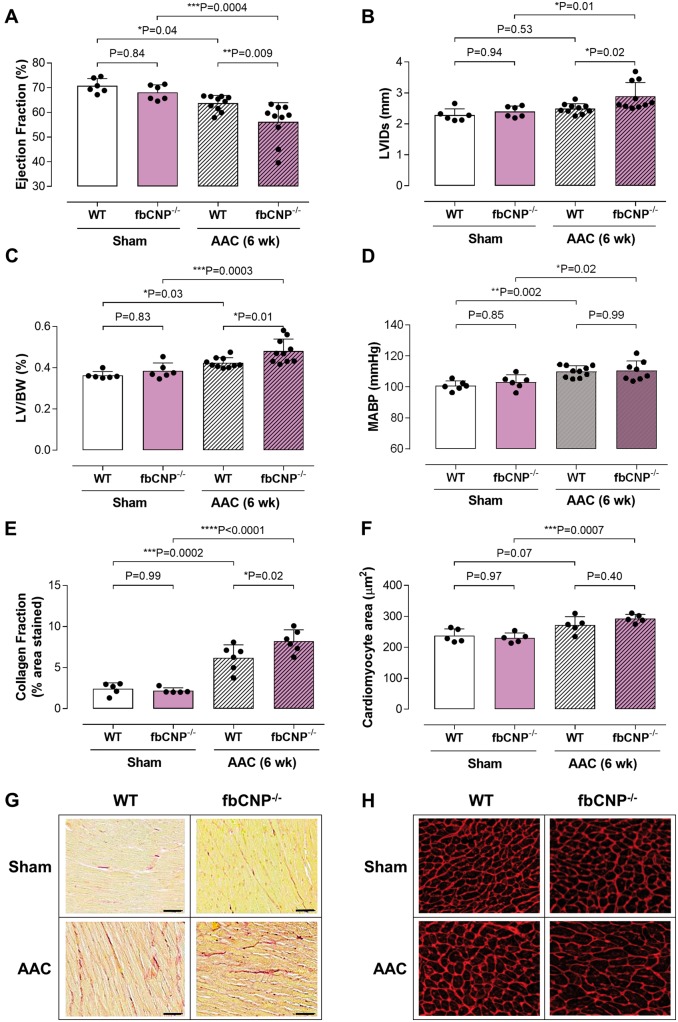
Fibroblast-specific deletion of CNP worsens the cardiac response to pressure-overload. Ejection fraction (*A*), left ventricular internal diameter at systole (LVIDs; *B*), left ventricle to body weight ratio (LV/BW; *C*), mean arterial blood pressure (MABP; *D*), fibrotic burden (collagen fraction; *E* and *G*; scale bar = 50 μm) and cardiomyocyte size (*F* and *H*) in WT and fbCNP^−/−^ animals exposed to 6 weeks abdominal aortic constriction (AAC). Data are presented as mean ± SD and analysed using one-way ANOVA with Šídák *post-hoc* test. Each statistical comparison undertaken has an assigned *P* value (adjusted for multiplicity).

The accentuated severity in NPR-C^−/−^ animals in the face of pressure overload (*Figure 4*) intimated that CNP from a separate cellular source might play a functional role in triggering cardioprotective NPR-C. One possibility is that cardiac fibroblasts fulfil this capacity.^27^ To explore this potential mechanism, we generated a fibroblast-specific CNP knockout line (fbCNP^−/−^). Whilst there was no basal cardiac phenotype in these mice (Supplementary material online, *Figure S7*) when exposed to pressure overload fbCNP^−/−^ animals also exhibited an exacerbated phenotype compared to WT littermates (albeit more modest than that seen in cmCNP^−/−^ or NPR-C^−/−^ mice; *Figure 7*). However, identical studies conducted in ecCNP^−/−^ animals suggest that endothelium-derived CNP plays little or no role in terms of cardioprotection during HF (Supplementary material online, *Figure S5*).

### Endothelium-derived C-type natriuretic peptide regulates coronary reactivity via natriuretic peptide receptor-C

**Figure 8:**
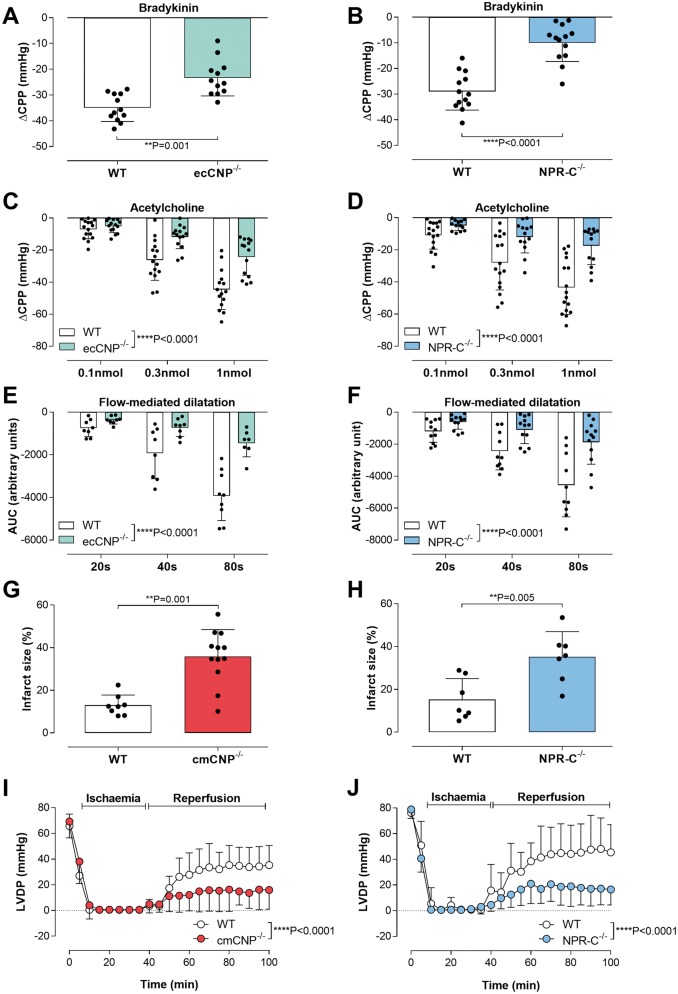
Endothelial CNP regulates coronary vascular reactivity and ischaemia/reperfusion injury via NPR-C. (*A* and *B*) Bradykinin (10 nmol), (*C* and *D*) acetylcholine (0.1–1 nmol), and (*E* and *F*) flow-mediated dilatation (zero flow for 20-80 s followed by reperfusion at 2 mL/min)—dependent decreases in coronary perfusion pressure (CPP) in isolated Langendorff hearts from WT, ecCNP^−/−^ and NPR-C^−/−^ mice. (*G* and *H*) Infarct size and (*I* and *J*) left ventricular developed pressure (LVDP) in isolated Langendorff hearts from WT, cmCNP^−/−^ and NPR-C^−/−^ mice subjected to 35 mins global ischaemia (zero flow) followed by 60 mins reperfusion (2 mL/min constant flow). Data are presented as mean ± SD and analysed using two-way ANOVA with Šídák *post-hoc* test (*C*, *D*, *E*, *F*, *I* and *J*) or Student's *t*-test (*A*, *B*, *G* and *H*).

Bradykinin (BK), acetylcholine (ACh), and flow-mediated dilatation (i.e. acute increases in shear stress) elicited endothelium-dependent decreases in coronary perfusion pressure (CPP; i.e. vasodilatation) in WT mice that were significantly impaired in ecCNP^−/−^ animals (*Figure 8*). However, responses to exogenous CNP and the direct-acting vasodilator sodium nitroprusside (SNP) were unchanged, indicating the deficit was of endothelial origin (Supplementary material online, *Figure S8*). Vasodilator responses to BK, ACh, and flow-mediated dilatation were correspondingly diminished in NPR-C^−/−^ hearts (*Figure 8*), as was the vasodilator response to exogenous CNP (Supplementary material online, *Figure S8*; although a residual drop in CPP persisted, likely due to activation of NPR-B^2,^^3^). Finally, release of CNP into the coronary effluent was markedly reduced in hearts from ecCNP^−/−^ mice in response to ACh (Supplementary material online, *Figure S8*).

**Take home figure:**
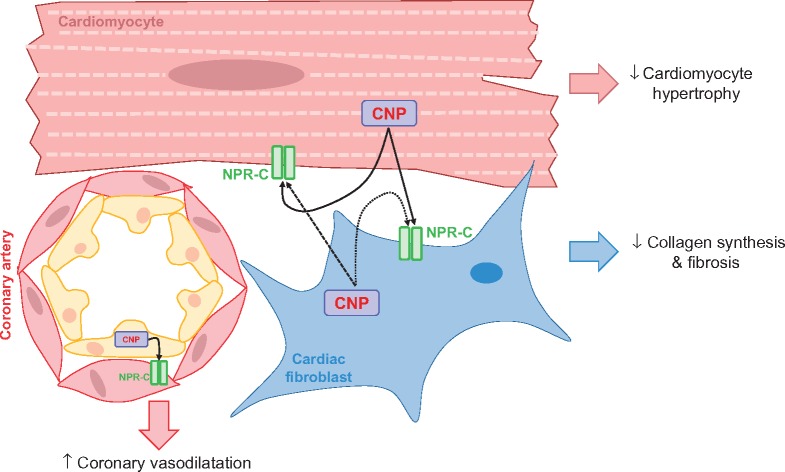
C-type natriuretic peptide (CNP) produced by multiple cell types within the heart acts in concert to reduce cardiac hypertrophy, cardiac fibrosis and improve coronary blood flow.

### Cardiomyocyte-derived C-type natriuretic peptide protects against ischaemia–reperfusion injury

Genetic ablation of cardiomyocyte-derived CNP resulted in a significantly greater infarct area and prolonged impairment in LV function following I/R injury (*Figure 8*). In contrast, hearts from ecCNP^−/−^ mice behaved in an ostensibly identical fashion to WT littermates (Supplementary material online, *Figure S8*).The phenotype in NPR-C^−/−^ animals recapitulated that observed in the cmCNP^−/−^ mice (*Figure 8*). These data suggest that a NPR-C-triggered pathway underpins the cardioprotection proffered by cardiomyocyte-derived CNP following I/R injury.

## Discussion

The present study tenders definitive evidence for major physiological and pathological roles for CNP in the regulation of cardiac structure and function. Cardiomyocyte-derived CNP has a subtle effect on basal cardiac function with a modest fall in HR in cmCNP^−/−^ mice (i.e. circadian rhythm and blood pressure independent). Published evidence points to a dual role of CNP and/or NPR-C in sinoatrial node conduction,^28^ HR (variability),^29^ and susceptibility to arrhythmia,^28^^,^^29^ perhaps via reductions in cardiac sympathetic transmission.^30^ Such actions of CNP may represent an important protective mechanism and potential therapeutic target in HF patients as damping sympathetic activity improves survival.^31^^,^^32^ Preliminary evidence that cardiomyocyte-derived CNP might play a more substantive role in the response to cardiac stress was provided by a serendipitous observation of early mortality in breeding females in which LV hypertrophy during pregnancy accommodates the needs of the foetus. Accordingly, irrespective of the precipitating stimulus (i.e. pressure overload or sympathetic hyperactivation), cmCNP^−/−^ mice fared worse with respect to several indices of cardiac structural and functional integrity in experimental HF; isolated cardiomyocytes from cmCNP^−/−^ animals also exhibited an exaggerated hypertrophic response *in vitro*. These findings corroborate the concept of CNP as a ‘cardiac natriuretic peptide’^25^ and extends the cohort of natriuretic peptides key to heart health and disease above and beyond ANP and BNP.

Subsequent studies verified the NPR signalling mechanism responsible for the cardioprotective influence of CNP. The phenotype of NPR-C^−/−^ mice following pressure overload mirrored that observed in cmCNP^−/−^ animals; indeed, if anything the severity was greater. It is possible this aggravated cardiac dysfunction is due to lack of complete deletion of CNP from cardiomyocytes in cmCNP^−/−^. However, this dichotomy also raises the possibility that alternate cellular sources of CNP might contribute to cardioprotection. Endothelium-restricted CNP deletion did not result in a worse phenotype in the face of pressure overload. However, an alternate hypothesis is that this supply might be from the cardiac fibroblast, since CNP is synthesized and secreted from these cells.^27^ In order to provide proof-of-concept to support this hypothesis, we developed a unique fibroblast-specific CNP null mutant and exposed these mice to pressure overload. Whereas basal cardiac functional parameters and mean arterial blood pressure were not significantly disturbed, fbCNP^−/−^ mouse did exhibit a modestly more severe phenotype following AAC. This finding supports the thesis that cardiac fibroblasts synthesize and release CNP in response to cardiac stress that complements the protective function of cardiomyocyte-derived CNP. This was corroborated by more molecular investigation in which ANP, βMHC, and Col1α1 were up-regulated, and SERCA-2 down-regulated, in all gene knockout strains, exemplifying common mechanisms underpinning the beneficial effects of CNP/NPR-C signalling. These observations fit well with previous work verifying that CNP directly inhibits collagen synthesis in cardiac fibroblasts.^27^ Finally, up-regulation of critical pro-fibrotic mediators, TGFβ and fibronectin, was observed in fbCNP^−/−^ and cmCNP^−/−^ mice, respectively; changes in both were found in NPR-C^−/−^ hearts. This finding suggests that to exert its maximal anti-fibrotic capacity, CNP release from both cardiomyocytes and cardiac fibroblasts is essential. However, the beneficial bioactivity of CNP in HF appears to be NOS independent.

Intriguingly, the detrimental response to cardiac stress was not recapitulated in NPR-B^−/−^ mice. Such data support the conclusion that NPR-C, rather than NPR-B, is the principal mechanism via which CNP exerts its cardioprotective effect. This fits well with recent data in a cardiomyocyte-specific NPR-B^−/−^ strain that exhibits a negligible intrinsic phenotype in response to pressure overload,^33^ diminished ventricular expression of NPR-B in HF patients (albeit not recapitulated herein),^7^ impaired sinoatrial conduction and aggravated atrial fibrosis in NPR-C^−/−^ mice,^28^ and a human NPR-C genetic variant that precipitates LV dysfunction.^34^ This concept is also corroborated herein by pharmacological delivery of CNP which completely reversed the structural and functional deficits associated with pressure overload in WT, but not NPR-C^−/−^, mice (although some recent reports have proposed a protective outcome following NPR-C deletion or antagonism in experimental HF^35^^,^^36^). The fact that CNP was able to restore heart morphology and contractility in WT mice exposed to pressure overload suggests that even in the presence of endogenous CNP it is possible to boost NPR-C-dependent signalling for therapeutic gain. In fact, this study reveals that ventricular specimens from HF patients and healthy controls have a predominant cardiomyocyte expression of NPR-C, whereas in failing hearts additional fibroblast localization is observed; such findings support the concept that CNP-dependent NPR-C activation on both cardiomyocytes and fibroblasts drives the cardioprotective actions of the peptide. Additionally, it is demonstrated, herein, that ventricular CNP expression is diminished in murine pressure overload and human HF, whereas NPR-C levels are augmented (at least in human ventricular tissue); this hints that pharmacological administration of CNP or NPR-C agonists may be even more efficacious in HF patients. However, there is a disconnect between these observations and previous studies reporting increased myocardial CNP release and plasma CNP levels in patients with HF.^17^^–^^19^ Whether this results from the short-term nature of the experimental model utilized herein, and that more chronic release of CNP (as an intrinsic protective mechanism) is required to sustain elevated plasma concentrations, remains to be clarified. It might also be hypothesized that endothelial CNP release, rather than myocardial, contributes predominantly to the higher circulating levels of the peptide in HF patients. Nevertheless, there was a significant increase in plasma BNP (and a trend in ANP) in cmCNP^−/−^ mice with HF, perhaps indicative of the exacerbated phenotype and/or an intrinsic mechanism compensating for loss of cardiomyocyte-derived CNP. Yet, an extensive literature supports the concept that both ANP and BNP exclusively exert their beneficial vascular (e.g. vasodilatation, diuretic) and cardiac (e.g. anti-hypertrophic) actions via activation of NPR-A since such responses are completely abrogated in NPR-A^−/−^ mice, either following global or cell-specific deletion.^37–41^ This suggests that NPR-C activation plays little or no role in any compensatory cardio- and/or vaso-protective roles of ANP and BNP, despite the fact that both peptides bind NPR-C.^12^ Moreover, this study provides evidence that natriuretic peptide levels are not significantly altered in NPR-C^−/−^ KO following pressure overload (mirroring measurements under physiological conditions^42^) ruling out an indirect effect (i.e. the clearance function) of NPR-C on the cardiac phenotype in these animals (albeit, if this were true, one would predict a better, not aggravated, outcome in mice lacking NPR-C). In contrast, augmentation of natriuretic peptide bioactivity is thought to underpin the efficacy of the dual neprilysin/angiotensin receptor blocker LCZ696 (Entresto) in HF^43^; indeed, since CNP is the most susceptible of the natriuretic peptides to neprilysin degradation^44^ it might be postulated that this member of the family would contribute the greatest cardioprotective influence.

C-type natriuretic peptide also plays a fundamental role in the maintenance of myocardial perfusion. Mice with endothelial-restricted deletion of CNP exhibit a sharp reduction in the responsiveness to endothelium-dependent dilators and shear stress in the coronary circulation. Moreover, this deficiency in accompanied by a significant decrease in the release of CNP from the coronary vasculature, substantiating the link between endothelium-derived CNP and coronary homoeostasis; such observations also dovetail well with shear stress as a key trigger for endothelial CNP release.^45^ In conduit vessels CNP-induced relaxation is NPR-B-dependent^10^ but in the resistance vasculature the importance of NPR-C in the vasoreactivity of CNP increases.^1^ This is illustrated by the normotensive phenotype of NPR-B^−/−^ mice^1^^,^^2^^,^^11^ vs. the hypertension in ecCNP^−/−^ animals.^1^^,^^2^ In the present study, coronary endothelium-dependent vasoreactivity and responsiveness to exogenous CNP were markedly blunted in NPR-C^−/−^ mice, implying that NPR-C activation primarily underpins the coronary actions of CNP. This clear delineation of a CNP/NPR-C signal transduction system adds significantly to the understanding of mechanisms underpinning coronary vascular homoeostasis, and is likely to have important implications for heart disease. For example, the ability of endothelium-derived CNP to regulate coronary vascular reactivity, coupled to its pronounced effect on leucocyte flux, platelet function and atheroma,^1^ suggests mimicking CNP bioactivity pharmacologically is likely to be an effective means by which to slow the progression of coronary artery disease. Moreover, coronary microvascular dysfunction is a hallmark of HF with preserved ejection fraction (HFpEF); promoting or recapitulating the bioactivity of endothelium-derived CNP might represent a new approach to reversing this issue as a disease-modifying therapy.

C-type natriuretic peptide/NPR-C signalling is also important in innate defence against I/R injury. However, in this context, it is cardiomyocyte, rather than endothelium, -derived CNP that appears key. This is perhaps surprising since the restoration of flow (i.e. reperfusion) should trigger the release of CNP from the coronary endothelium (as demonstrated above). Yet, genetic ablation of endothelium-derived CNP does not affect outcome. Mirroring observations in the coronary vasculature, genetic deletion of NPR-C recapitulates the unfavourable phenotype in hearts from cmCNP^−/−^ following I/R. These data reveal a novel intrinsic capacity of the myocardium to protect itself against I/R injury via release of cardiomyocyte-derived CNP and autocrine activation of NPR-C (a role for NPR-B has also been previously reported^13^^,^^46^). Indeed, the cardioprotective potential of CNP/NPR-C signalling is likely to be underestimated in the present study because experiments were conducted in the absence of blood perfusion; the well-established pathological roles of leucocytes and platelets in MI should also be dampened by NPR-C activation,^1^ reducing further the extent of damage. Further investigation is warranted to define the underlying salutary pathways. It is well established that K_ATP_ channel opening is protective in I/R injury and members of the K_ATP_ family are opened by the βγ-subunits of G_i_-coupled NPR-C,^21^^,^^47^ suggesting the beneficial effect of CNP against I/R injury might be mediated via such a mechanism, as we have described *ex vivo.*^21^ In support of this concept, elevated *Nppc* and *Npr3* mRNA expression is found in ischaemic hearts.^18^

In sum, herein, we define CNP of cardiomyocyte, endothelium, and cardiac fibroblast origins as a key player in the physiological maintenance of coronary vascular homoeostasis and host response to cardiac stress. These cardioprotective functions of CNP are mediated predominantly via activation of G_i_-coupled NPR-C, identifying a new target in the fight against ischaemic cardiovascular disorders and HF.

## Funding

British Heart Foundation Programme Grant [RG/16/7/32357 to A.J.H.], a BHF PhD studentship [FS/13/58/30648 to S.M.C.] and procurement of human heart tissue enabled by grants from the NHLBI Institute of the US National Institutes of Health [HL089847 and HL105993 to K.B.M.].

**Conflict of interest:** A.J.H. is a scientific advisory board member for Palatin Technologies Inc. and is a named inventor on a patent describing NPR-C ligands.

## Supplementary Material

ehz093_Supplementary_Data
