# Supplementary material for: C-type natriuretic peptide co-ordinates cardiac structure and function
Source: Eur Heart J. 2019 Mar 21;41(9):1006–20. doi: 10.1093/eurheartj/ehz093 (PMC7068173; doi:10.1093/eurheartj/ehz093)
Supplement: ehz093_Supplementary_Data [file ehz093_supplementary_data.pdf]

SUPPLEMENTARY DATA

Moyes et al. 2019

SUPPLEMENTARY METHODS

All studies conformed to the UK Animals (Scientific Procedures) Act of 1986 and had approval from a local Animal Welfare and Ethical Review Body.

Generation of cell-restricted CNP knock-out mice

Endothelial cell-specific CNP knockout (ecCNP$^{-/-}$) were developed in house[1], global NPR-B$^{-/-}$ (*Peewee*)[2] were the kind gift of Prof. S. Camper (University of Michigan), and global NPR-C$^{-/-}$[3] were the kind gift of Prof. O. Smithies (University of North Carolina?). Cardiomyocyte-specific CNP deletion (cmCNP$^{-/-}$) was achieved by crossing CNP$^{flox/flox}$ animals[1] with mice expressing an alpha myosin heavy chain (αMHC)-driven Cre-recombinase (kind gift of Prof. M. Schneider, Imperial College London). To generate fibroblast–specific CNP null mutants (fbCNP$^{-/-}$), CNP$^{flox/flox}$ animals were mated with a tamoxifen-inducible Collagen 1α2-Cre strain (Jax labs, USA). In this case, gene deletion was achieved by injecting tamoxifen (40mg/kg/day [Sigma, Poole, UK], dissolved in sunflower oil containing 10% absolute ethanol; i.p.) for 5 consecutive days (to both WT and fbCNP$^{-/-}$ mice). Animals that were CNP$^{+/+}$ and positive for the Cre transgene were denoted as WT. Littermate controls (WT) were used throughout.

Genotyping

Genomic DNA was prepared from ear biopsies for analysis by polymerase chain reaction (PCR) using standard cycling parameters using the forward and reverse primers stated in ***Supplementary Table 4***, as we have described previously[4]. CNP$^{flox/flox}$ animals were identified by a band at 956bp whereas non-floxed animals have a band position at 842bp, Tie[2]-Cre expressing mice display a band at 512bp, αMHC-Cre at 990bp, and Col1α2-Cre at 700bp.See table 4 as some of these primer sequences were incorrect. NPR-C KO mice were identified by a band at 413bp with WT at 250bp (***Supplementary Figure 1***). NPR-B$^{-/-}$ mice were genotyped using primers directed to a four nucleotide deletion in exon 3 of the *Npr2* gene (deletion of CTGC generates a premature stop codon at position 313[2]).

Real-time (q)PCR analysis of CNP expression

To confirm the selective deletion of CNP from cardiomyocytes, neonatal cardiomyocytes from cmCNP$^{-/-}$ and WT littermates were isolated and cultured using the Pierce Primary Cardiomyocyte Isolation Kit (Thermo Scientific,USA). Cardiomyocytes in each well were collected with 350μL of buffer RLT (Qiagen RNeasy Mini Kit, Germany) containing 1% β-mercaptoethanol into a 1.5mL tube. The collected cells were snap-frozen immediately with liquid nitrogen and stored at -80°C for RNA extraction. Total RNA was extracted from cardiomyocytes and organs using a RNeasy Fibrous Tissue Mini Kit (Qiagen, Germany) according to manufacturer instructions. 250ng RNA from each cell sample and 1000ng from each tissue sample was converted to cDNA using the QuantiTect® Reverse Transcription Kit (Qiagen) according to manufacturer instructions. The cDNA products were stored at -20°C for qPCR analysis. Quantitative real-time PCR was performed using the Quantitect SYBR green kit (Qiagen). The cardiomyocyte cDNA samples were diluted 1:2 and the samples from organ tissue were diluted 1:20 resulting in cDNA concentrations of 6.25ng/μL and 50ng/μL, respectively. 2μL of the cDNA sample was then added to 8μL of the qPCR master mix. Primers were designed that spanned *Nppc* gene exons 2 & 3 (10μM; 5'-AAAAGGGTGACAAGACTCCAGGCAG-3' and 5'-GGTGTTGTGTATTGCCAGTA-3'). Results were analyzed using the ABI Prism 7900HT software package SDS 2.4 (Advanced Biotechnologies Ltd). CNP mRNA levels were normalized to β-actin and RPL-19 (internal controls) for each sample. The relative quantification of CNP expression between cmCNP$^{-/-}$ and WT were determined by the $2^{-\Delta\Delta CT}$method, where ΔΔCT is the fold change relative to WT levels.

Expression of CNP mRNA was significantly reduced (~60%) in cardiomyocytes isolated from cmCNP$^{-/-}$ mice compared to WT littermates (***Supplementary Figure 1***). Further analysis indicated that the isolated cardiomyocyte population was approximately 80% pure (ratio of Troponin T-positive to DAPI-positive cells), resulting in ~80% efficiency in CNP deletion. In addition, CNP mRNA expression was significantly reduced in whole hearts from cmCNP$^{-/-}$ mice, but equivalent in all other tissues/organs examined (***Supplementary Figure 1***). Deletion of the LoxP sites was confirmed by PCR in which an excised DNA band was only observed in cmCNP$^{-/-}$ hearts, and not in WT or cmCNP$^{-/-}$ liver or lung (***Supplementary Figure 1***). Deletion of CNP from (cardiac) fibroblasts was confirmed by isolating and culturing these cells from hearts of WT and fbCNP$^{-/-}$ animals and determining CNP mRNA expression levels (***Supplementary Figure 1***). These studies revealed an approximate 75% deletion of CNP from cardiac fibroblasts, but without a significant reduction in expression in whole organs

(***Supplementary Figure 1***). In concert, these data confirm efficient, exclusive removal of the *Nppc* gene from the cardiomyocyte or (cardiac) fibroblast.

***Ex-vivo* functional assessment of coronary vascular reactivity**

Coronary reactivity was evaluated in murine hearts set-up in Langendorff mode. Animals were injected with heparin (100 µL; i.p.) prior to anesthesia with isoflurane (3 % in O_2). Following opening of the chest cavity, the heart was quickly excised and placed into ice-cold Krebs-Henseleit buffer (composition: NaCl 118.5 mM; KCl 4.7 mM; $MgSO_4$ 2.4 mM; KH_2PO_4 1.2 mM; Glucose 11.9 mM; $NaHCO_3$ 25.0 mM; $C_3H_3NaO_3$ 2.0 mM; $CaCl_2$ 1.7 mM). The heart was subsequently mounted and an intraventricular balloon inserted into the LV through the left atrium. The balloon was inflated to a total volume of 24µL (female) or 28 µL (male). The perfusion system was maintained at a constant flow rate of 2 mL/min via a peristaltic pump (Miniplus 3; Gilson, Middleton, USA); a constant temperature (37°C) and pH (7.4) was maintained and gassed with 95% O_2/ 5% CO_2. Coronary perfusion pressure (CPP), left ventricular developed pressure (LVDP), and heart rate (HR) were recorded by inline transducers (Hugo Sachs Electronic, March, Germany) connected to a Power Lab 4/30 data acquisition system (ADInstruments, Bella Vista, Australia). After 10mins of stabilization, the perfusion solution was switched to Krebs buffer solution containing the NOS inhibitor N^G-nitro-L-arginine methylester (L-NAME; 300µM). Hearts that did not show a >50mmHg increase in CPP in the presence of L-NAME (indicative of a functional endothelium) were discarded. Acute changes in CPP were recorded in response to bolus injections of the endothelium-dependent vasodilators bradykinin (BK; 10 nmol) and acetylcholine (ACh; 0.1-1 nmol), CNP (10 nmol) and the NO-donor sodium nitroprusside (SNP; 1 nmol). 10 µL of each drug was administered with a Hamilton syringe into the aortic cannula and each injection was separated by at least 5 min in order for the CPP to return to baseline before adding the next dose or drug. Hearts were also exposed to three periods of zero flow (20, 40 and 80 seconds) and the resulting response to restoration of flow (flow-mediated dilatation; shear stress-induced release of endothelium-borne vasodilators[5]) was assessed using area under the curve (AUC). Occlusion periods were at least 5mins apart, allowing the CPP to return to baseline. The extent of vasodilatation was quantified by percentage change in CPP. Additionally, following administration of 1 nmol ACh, 10ml (5 min of perfusion) of coronary effluent was collected from the apex of the hearts and immediately frozen in liquid nitrogen for CNP bioassay.

Ischemia-reperfusion injury

In some studies, cardiac function was investigated following ischemia-reperfusion (I/R) injury. This was induced by global cessation of flow for 35 min then reperfusion for 60 min. After reperfusion, the hearts were frozen at -20°C for 10 min and then immediately sliced with a scalpel into 1mm thick sections perpendicular to the long-axis of the heart. The slices were subsequently incubated with 1% triphenyl tetrazolium chloride (TTC) made in PBS at 37°C for 15 min. The heart sections were photographed with a scanner on both sides and the infarcted area (white) was measured in Image J and expressed as a percentage of total ventricular area.

CNP bioassay

CNP was extracted from effluent samples using C18 columns (Phoenix Pharmaceuticals, Karlsruhe, Germany). The eluants were then evaporated to dryness by a centrifugal concentrator (Speedvac, Thermo Scientific, US) overnight. Samples were reconstituted in 125μL assay buffer and a CNP EIA was performed according to the manufacturer's instructions (Phoenix Pharmaceuticals).

Pressure overload-induced HF

Pressure-overload LVH and cardiac dysfunction were induced by performing abdominal aortic constriction (AAC) at the suprarenal level. Male WT, ecCNP$^{-/-}$, cmCNP$^{-/-}$, fbCNP$^{-/-}$ & NPR-C$^{-/-}$ mice (21–23 g; offspring of heterozygote parents to enable use of corresponding WT littermate controls) were anesthetized (1.5% isoflurane in O_2), body temperature maintained at 37°C and the analgesic buprenorphine (0.1 mg/kg; s.c.). An incision was made in the abdominal cavity, and the abdominal aorta was separated from the surrounding tissue at the suprarenal level. Aortic constriction was performed by tying a 4-0 surgical thread against a 25-gauge needle between the superior mesenteric and renal arteries. This produces a 30% constriction of the luminal diameter[6]. For sham operations, the 4-0 surgical thread was passed under the aorta and removed without tying it against the needle. In some studies, WT mice undergoing AAC were administered (a) CNP (0.2mg/kg/day; s.c. by osmotic minipump, initiated 3 weeks following AAC surgery and maintained throughout the study) and/or (b) the NOS inhibitor L-N^{G}-nitroarginine methylester (L-NAME; 100 mg/kg/day via the drinking water, initiated 3 days prior to AAC surgery and maintained throughout the study). Plasma natriuretic peptide concentrations were

determined by specific enzyme immunoassay (Phoenix Pharmaceuticals Inc., Karlsruhe, Germany) as previously described[1].

Chronic sympathetic activation-induced HF

Male WT, $cmCNP^{-/-}$ and $NPR\text{-}B^{-/-}$ mice (24-25 g;) were infused subcutaneously with isoproterenol (20 mg/kg/day, 7 days; Sigma-Aldrich, Poole, UK) via osmotic mini-pumps (model 1002, Alzet, Cupertino, CA). Saline containing 0.5% ascorbic acid was used as the solvent for isoproterenol to avoid catecholamine oxidation over time.

In vivo cardiac functional assessments

Echocardiograms were obtained from anesthetized mice at baseline and 6 weeks for the AAC model or at baseline and 1 week following mini-pump implantation for the isoproterenol model. *In vivo* cardiac morphology and function were assessed by M-mode echocardiography using a VisualSonics Vevo 770 echocardiographic system and a 30 MHz transducer. Mice were anesthetized (1.5% isoflurane in O_2) and body temperature maintained at 37°C. LV internal diameter (LVID), and LV posterior wall thicknesses (LVPW) at diastole (d) and systole (s) were measured from short-axis M-mode images. LV ejection fraction (EF%) was calculated as follows: $LVEF\% = [(LVIDd)^3 – (LVIDs)^3]/(LVIDd)^3 × 100$; LV fractional shortening (FS%) was calculated as follows: $LVFS\% = (LVIDd – LVIDs)/LVIDd × 100$. Values were averaged from 3 beats. Electrocardiogram (ECG), heart rate (HR), and respiration rate were also recorded.

Histology, staining and imaging

For murine studies, the isolated left ventricles were cut transversely below the mitral valves, fixed in 10% formalin for 24h, then stored in 70% ethanol before embedding in paraffin wax and sectioning.

Wheat germ agglutinin fluorescent staining

In order to measure the ventricular myocyte size, the tissue slides were stained with a fluorescent cell membrane antibody, wheat germ agglutinin alexafluor 647 (1:500; Molecular Probes, Invitrogen, UK) and mounted with Prolong gold DAPI mountant as per standard immunohistochemistry protocols. Images were taken on a Zeiss 710 confocal microscope and the cardiomyocyte size analyzed with Image J (National Institutes of Health).

Cardiomyocyte size was estimated with investigator blinded to treatment type and as an average of >1000 cells per heart.

Picrosirius red staining

Tissue slides were dewaxed, rehydrated and stained using a Picrosirius Red Stain kit following the manufacturer's instructions (Polysciences, Inc. Warrington, PA, USA. A Nikon Eclipse TS100 microscope (Nikon UK Limited, Surrey, UK) was used to capture images of the stained slides. Images were analyzed by threshold analysis using Image J (National Institutes of Health) with investigator blinded to treatment type.

Immunofluorescence analysis

Immunofluorescence was used to localize NPR-C (anti-NPR-C, ab37617, 1:100; Abcam) in the cardiomyocytes (troponin T; MS-295-P0, 1:100; ThermoFisher Scientific, UK) and cardiac fibroblasts (vimentin; 919101, 1:1000; Biolegend) Following routine removal of paraffin and antigen retrieval using citrate-based solution (pH6.0, Vectorlabs, UK), human ventricular sections were permeabilized for 10 min in 0.2% Triton X-100, in blocking solution (10% goat serum/1% bovine serum albumin (BSA) in PBS) at room temperature for 1 h and probed with the primary antibodies diluted in the blocking solution overnight at 4°C. After primary antibody incubation, sections were washed with PBS for three times and incubated with appropriate Alexa Fluor-coupled secondary antibodies (1:400, Molecular Probes) for 1 h at room temperature. To counteract auto-fluorescence, sections were incubated in 0.3% Sudan black. Nuclei were counterstained with DAPI. Sections were thoroughly washed with PBS before mounting them with ProLong mounting media (ThermoFisher Scientific). Finally, coverslips were sealed with nail polish.

Primary cardiomyocyte isolation and culturing

The Pierce primary cardiomyocyte isolation kit (Thermo Scientific, Loughborough, UK) was used. Neonatal hearts from WT and cmCNP$^{-/-}$ mice were dissected from 2-3 day old mice and placed in 500μL ice cold HBSS. The hearts were then minced into 1-3mm^3 pieces in a petri dish and transferred to 1.5mL tubes. The tissues were washed twice with 500μL ice cold HBSS to remove blood. 200μL of Cardiomyocyte Isolation Enzyme 1 (with papain) and 10μL of Cardiomyocyte Isolation Enzyme 2 (with thermolysin) were then added, mixed gently and incubated in a 37°C incubator for 30-35 min. The enzyme solution was then removed gently and the tissue washed twice with 500μL ice cold HBSS. Subsequently, 0.5 mL of

complete DMEM (containing 10 % FBS and 1 % Pen-Strep) was added to each tube and pipetted up and down 25-30 times using a sterile 1 mL pipette tip to break up the tissue into a single cell suspension. 1mL of complete DMEM was then added to each tube to bring the total volume to 1.5 mL. The cell suspension in each tube was seeded into two separate wells of a gelatine (0.1 %) pre-coated 12-well plate, i.e. 0.75 mL into each well, and topped up with 0.25 mL of complete DMEM to bring the total volume to 1 mL. The plates were then incubated at 37°C in a 5%CO_2 incubator for 24 h. The medium was replaced with fresh complete DMEM containing Cardiomyocyte Growth Supplement provided by the kit. The cultures were then further incubated for 3 days for the cells to become confluent. The cells were then serum starved for 24 h before Angiotensin (Ang) II (1 μM) treatment. Light microscopy images of beating cardiomyocytes were taken at 0 h (baseline), 24 h and 48 h. Image J were used to analyze the cardiomyocyte size. The purity of the cardiomyocyte population was determining the percentage of cells which stained positive for the cardiomyocyte-specific marker Troponin T (ab8295; 1:200; Abcam, Cambridge, UK) in comparison to the total cell population (DAPI-positive; 1 μg/mL; Sigma).

Primary cardiac fibroblast isolation and culturing

Hearts from adult WT and fbCNP$^{-/-}$ animals were isolated and placed into a falcon tube containing sterile ice-cold PBS and washed twice to remove any blood. The hearts were then placed on a sterile surface, cut into 1 mm pieces, and transferred into a T75 flask. Tissues were spread around the flask using a cell scraper and left for 25 min at room temperature. 20 mL of media containing DMEM, 15% FBS and 1% pen/strep were applied to the top side of the flask and slowly placed the flask flat without disturb the tissues on the base of the flask. This was then incubated in a 37°C incubator for 7 days to allow fibroblasts to grow out from the tissue. Once the cells approached confluency, the old media was removed and tissues were washed with sterile PBS twice. 3 mL trypsin was added into the flask for 5 min to detach the cells. An equal amount of FBS was then added and centrifuged at 1000 rpm for 5 min. The supernatant was discarded and the cell pellet was transferred into a new T75 flask and incubated in 20 mL media (DMEM, 10% FBS, 1% pen/strep) for 10 days or until confluence was reached. Cells were stained with the fibroblast marker vimentin (D21H3 XP Rabbit mAb Alexa Fluor 488 Conjugate; 1;1000; New England Biolabs) to confirm that a pure population of cardiac fibroblasts had been isolated.

Quantitative RT-PCR & immunoblotting

Whole hearts were snap frozen, broken down using a pestle and mortar and then homogenized using QIAshredder technology. RNA was extracted using a Fibrosis Tissue extraction kit (as above; Qiagen) and quantified using a NanoDrop spectrophotometer (ThermoFisher Scientific, MA, USA). 1 µg of RNA was converted to cDNA by reverse transcription (High Capacity RNA-to-cDNA Kit; Applied Biosystems, Life technologies Ltd, UK). Specific primers for hypertrophic or fibrotic markers and housekeeping genes RLP-19 and β-actin (300 nM; detailed in ***Supplementary Table 1***) were added to cDNA template and SyBr Green quantitative PCR mix (Quantitect Sybr green kit, Qiagen, UK). 2.5 ng of cDNA from each sample was amplified using quantitative real-time PCR over 40 cycles (initial denaturation: 10 min at 95°C; cycling: 45 cycles, 10 s at 95°C, 15 s at 57°C, and 5 s at 72°C; melt: 68-90°C). mRNA expression was analyzed by expressing the cycle threshold (Ct) value as $2^{-\Delta\Delta Ct}$, relative to the levels of *RPL19* for tissue samples and *GAPDH* for cells, and further normalized as a fold change to control treatments.

Pro-hypertrophic, pro-fibrotic marker expression and/or phosphorylation was assessed by immunoblot using standard protocols and the following specific antibodies: anti-Gα(q) (D5V1B; 1:1000; Cell Signalling Technology), anti-phospho-GSK-3β (Ser9; 1:1000; Cell Signalling Technology), GSK-3β (27C10; 1:1000; Cell Signalling Technology), anti-Smad2 (1:1000; Abcam), anti-phospho-Smad2 (1:500; Abcam), anti-Smad3 (1:1000; Abcam), anti-phospho-Smad3 (1:1000; Abcam), anti-p44/42 (ERK1/2; L34F12; 1:1000; Cell Signalling Technology), and anti-phospho-p44/42 (Thr202/Tyr204; 1:1000, Cell Signalling Technology). Phosphoproteins were normalised to respective total protein expression, and all other proteins normalised to housekeeping GAPDH (1:50,000; ThermoFisher Scientific).

Human myocardial tissue

Procurement of human myocardial tissue was performed under protocol ethical regulations and approved by Institutional Review Boards at the University of Pennsylvania and the Gift-of-Life Donor Program (Pennsylvania, USA). Failing human hearts were procured at the time of orthotropic heart transplantation at the Hospital of the University of Pennsylvania following informed consent from all participants. The aetiology of these samples was as follows: ischaemic cardiomyopathy (6 of 11) and idiopathic dilated cardiomyopathy (5 of 11); all had been diagnosed with HFrEF. Non-failing hearts were obtained at the time of organ harvesting from cadaveric donors. In all cases, hearts were arrested *in situ* using ice-cold cardioplegia solution and transported on wet ice. Whole hearts and dissected left ventricle cavity were weighed to determine levels of hypertrophy.

Transmural myocardial samples were dissected from the mid left ventricular free wall below the papillary muscle.

SUPPLEMENTARY FIGURES

Supplementary Figure 1. Generation of cardiomyocyte (cmCNP$^{-/-}$) and fibroblast (fbCNP$^{-/-}$) -specific CNP$^{-/-}$ mouse lines. Targeting strategy (***A***) and confirmation of cell-specific deletion of CNP in cmCNP$^{-/-}$ (***B***) and fbCNP$^{-/-}$ (***C***) animals. Data are presented as mean ± SD and analysed using one-way ANOVA with Šídák post-hoc test (upper panels, ***B*** & ***C***; adjusted for multiplicity) or Student's t-test (lower panels, ***B*** & ***C***; unadjusted for multiplicity). Each statistical comparison undertaken has an assigned P value.

Supplementary Figure 2. Successive pregnancies cause progressive cardiac dysfunction and mortality in cmCNP heterozygous female mice. Survival (***A***), left ventricular internal diameter at systole (***B***), and ejection fraction (***C***) in WT and cmCNP$^{+/-}$ female breeders following sequential pregnancies. Data are presented as mean ± SD and analysed using two-way ANOVA with Šídák post-hoc test. Each statistical comparison undertaken has an assigned P value (adjusted for multiplicity; n=6).

Supplementary Figure 3. **Global deletion of NPR-B does not worsen the cardiac response to pressure overload.** Ejection fraction (***A***), left ventricular internal diameter at systole (LVIDs; ***B***), left ventricle to body weight ratio (LV/BW; ***C***) and fibrotic burden (collagen fraction; ***D & E***; scale bar = 50μm) in WT and NPR-B$^{-/-}$ animals exposed to 7 days isoproterenol (ISO; 20mg/kg/day). Data are presented as mean ± SD and analysed using one-way ANOVA with Šídák post-hoc test. Each statistical comparison undertaken has an assigned P value (adjusted for multiplicity).

Supplementary Figure 4. Plasma natriuretic peptide levels are unchanged following in cmCNP$^{-/-}$ and NPR-C$^{-/-}$ mice following AAC. Plasma concentrations of (***A***) ANP, (***B***) BNP and (***C***) CNP in WT, cmCNP$^{-/-}$ and NPR-C$^{-/-}$ animals exposed to 6 weeks abdominal aortic constriction (AAC). Data are presented as mean ± SD. Data are presented as mean ± SD and analysed using Student's t-test. Each statistical comparison undertaken has an assigned P value (unadjusted for multiplicity).

Supplementary Figure 5. Neither endothelial-specific deletion of CNP nor inhibition of NO synthase alter the cardiac response to pressure overload. Ejection fraction (***A & E***), left ventricular internal diameter at systole (LVIDs; ***B & F***), left ventricle to body weight ratio (LV/BW; ***C & G***), and mean arterial blood pressure (MABP; ***D & H***) in WT and ecCNP$^{-/-}$ animals (***A-D***) or WT mice treated with CNP (0.2mg/kg/day) in the absence and presence of the NOS inhibitor L-NAME (100 mg/kg/day via the drinking water, initiated 3 days prior to AAC surgery and maintained throughout the study; ***E-H***) exposed to 6 weeks abdominal aortic constriction (AAC). Data are presented as mean ± SD. Data are presented as mean ± SD and analysed using one-way ANOVA with Šídák post-hoc test. Each statistical comparison undertaken has an assigned P value (adjusted for multiplicity).

Supplementary Figure 6. Cell-specific deletion of CNP, and global deletion of NPR-C, alters the expression of common hypertrophic and fibrotic gene markers/pathways. (***A***) Relative mRNA expression of a panel of biomarkers and/or drivers of cardiac hypertrophy and fibrosis altered in hearts from WT, cmCNP$^{-/-}$, fbCNP$^{-/-}$ and NPR-C$^{-/-}$ animals exposed to 6 weeks abdominal aortic constriction (AAC). (***B***) Venn diagram summarising the profile of gene and/or protein expression between genotypes (with additional targets unaltered by genotype depicted externally). Data are presented as mean ± SD and analysed using one-way ANOVA with Šídák post-hoc test. Each statistical comparison undertaken has an assigned P value (adjusted for multiplicity).

Supplementary Figure 7. Fibroblast-specific deletion of CNP does not alter basal cardiac structure or function. Left ventricular internal diameter at systole (LVIDs; ***A***), left ventricular posterior wall diameter at systole (LVPWs; B), ejection fraction (***C***), left ventricle to body weight ratio (LV/BW; ***D***), intraventricular septum diameter at systole (IVSs; ***E***), fractional shortening (***F***), mean arterial blood pressure (MABP; ***G***) and heart rate (***H***) in WT and fbCNP$^{-/-}$ animals. Data are presented as mean ± SD and analysed using Student's t-test. Each statistical comparison undertaken has an assigned P value (unadjusted for multiplicity).

Supplementary Figure 8. Endothelial CNP regulates coronary vascular reactivity but does not contribute to ischaemic/reperfusion injury. (***A & B***) CNP and (***C***) sodium nitroprusside –dependent decreases in coronary perfusion pressure (CPP) in isolated Langendorff hearts from WT, ecCNP$^{-/-}$ and NPR-C$^{-/-}$ mice. (***D***) Release of CNP from isolated Langendorff hearts from WT and ecCNP$^{-/-}$ animals. (***E***) Infarct size and (***F***) left ventricular

developed pressure (LVDP) in isolated Langendorff hearts from WT and ecCNP$^{-/-}$ mice subjected to 35 mins global ischaemia (zero flow) followed by 60 mins reperfusion (2 ml/min constant flow). (***G***) Representative 2,3,5-triphenyltetrazolium chloride (TTC)-stained heart sections illustrating the degree of infarct (white area) in the hearts from WT, ecCNP$^{-/-}$, cmCNP$^{-/-}$ and NPR-C$^{-/-}$ animals. Data are presented as mean ± SD and analysed using Student' t-test (***A***-***E***; unadjusted for multiplicity) or two-way ANOVA with Šídák post-hoc test (***F***; adjusted for multiplicity; n=12). Each statistical comparison undertaken has an assigned P value.

SUPPLEMENTARY TABLES

Target	Sequence (5'→3')
ANP	Forward: GGATTTCAAGAACCTGCTAGACC<br>Reverse: GCAGAGCCCTCAGTTTGCT
αMHC	Forward: GCCCAGTACCTCCGAAAGTC<br>Reverse: GCCTTAACATACTCCTCCTTGTC
βMHC	Forward: ACTGTCAACACTAAGAGGGTCA<br>Reverse: TTGGATGATTTGATCTTCCAGGG
SERCA2a	Forward: TGGAACCTTTGCCGCTCATTT<br>Reverse: CAGAGGCTGGTAGATGTGTT
TGF-β1	Forward: TCAGACATTCGGGAAGCAGT<br>Reverse: GCCCTGTATTCCGTCTCCTTG
Col1α1	Forward: TCTGACTGGAAGAGCGGAGAG<br>Reverse: AGACGGCTGAGTAGGGAACA
Fibronectin	Forward: CCGGTGGCTGTCAGTCAGA<br>Reverse: CCGTTCCCACTGCTGATTTATC
MMP-2	Forward: GACAAGTTCTGGAGATACAATGAAGTG<br>Reverse: CAGGTTATCAGGGATGGCATTC
TIMP-2	Forward: GATTCAGTATGAGATCAAGCAGATAAAGA<br>Reverse: GCGAGACCCCGCACACT
CNP	Forward: AAAAGGGTGACAAGACTCCAGGCAG<br>Reverse: GGTGTTGTGTATTGCCAGTA
CNP (human)	Forward: TACAAAGGAGCCAACAAGAAGG<br>Reverse: AAAGATGACCTCAGCACAACG
NPR-B	Forward: CCCTGTGCCTTTGACTTGGA<br>Reverse: GCAACAACATTTCCCAGCGA
NPR-B (human)	Forward: ACGGGCGCATTGTGTATATC<br>Reverse: GGGCTCTTATCAGCAGACGA
NPR-C	Forward: GGGGGTCCACGAGGTTTTTC<br>Reverse: CTCCACGAGCCATCTCCGTA
NPR-C (human)	Forward: TTGCACACGTCCATCTACAGT<br>Reverse: CTCTTCCATGAGCCATCTCCATA

RPL-19	Forward: GCTTGCCTCTAGTGTCCTCC<br>Reverse: TTGGCGATTTCATTGGTCTCA
β-Actin	Forward: GGCTGTATTCCCCTCCATCG<br>Reverse: CCAGTTGGTAACAATGCCATG

Supplementary Table 1. Primer sequences used to assess hypertrophic/fibrotic biomarkers and CNP-driven signalling pathways

ECG Parameters	WT	cmCNP$^{-/-}$
RR interval (ms)	109.3 ± 3.4	114.0 ± 3.8**
PR interval (ms)	35.0 ± 1.1	36.0 ± 1.4
P duration (ms)	11.1 ± 0.8	10.6 ± 1.0
QRS interval (ms)	9.2 ± 0.3	9.7 ± 0.5
QTc (ms)	70.6 ± 5.7	70.0 ± 1.0
QA interval (ms)	13.5 ± 0.5	14.7 ± 0.5*
SDNN (ms)	5.2 ± 0.6	6.8 ± 0.6
LF/HF ratio	1.7 ± 0.2	1.5 ± 0.2

Supplementary Table 2. Electrocardiogram parameters in WT and cmCNP$^{-/-}$ mice. Data are expressed as mean ± SD. *P<0.05, **P<0.01 vs. WT. n=8.

Genotype	Model	Plasma [CNP] (pg/ml)	P value
WT	AAC (6 wk)	17.3 ± 1.65	-
WT	AAC + L-NAME	20.0 ± 5.22	P=0.99<br>v WT AAC (6 wk)
WT	AAC + CNP	107.4 ± 43.13	P=0.06<br>v WT AAC (6 wk)
WT	AAC + L-NAME + CNP	166.7 ± 50.89	**P=0.0004<br>v WT AAC + L-NAME
NPR-C$^{-/-}$	AAC (6 wk)	21.3 ± 5.49	-
NPR-C$^{-/-}$	AAC + CNP	172.7 ± 36.61	P=0.36<br>v WT AAC + CNP<br>***P=0.0006<br>v NPR-C$^{-/-}$ AAC (6 wk)

Supplementary Table 3. Plasma CNP concentrations. Data are expressed as mean ± SD. P<0.05, **P<0.01 vs. WT. n=5-6.

Strain	Sequence (5'→3')
CNP$^{flox/flox}$	Forward: CCTTTATGCCAAGAGAACTTCCAGGAGG<br>Reverse: TCCTTCCTGACTTCCTTCTGCTCTCTATCC

Tie2-Cre	Forward: CCCTGTGCTCAGACAGAAATGAG<br>Reverse: CGCATAACCAGTGAAACAGCATTGC
αMHC-Cre	Forward: CCAATTTACTGACCGTACACC<br>Reverse: GTTTCACTATCCAGGTTACGG
Col1α2-Cre	Forward: ATCCGAAAAGAAAACGTTGA<br>Reverse: ATCCAGGTTACGGATATAGT
NPR-B$^{-/-}$	Forward: TCACCCCGAGTCCTGTTTC<br>Reverse: TCCTCGTAGCCTGTTCTGACC
NPR-C$^{-/-}$	Forward: CTTGGATGTAGCGCACTATGTC<br>Reverse: CACAAGGACACGGAATACTC<br>Neo reverse: ACGCGTCACCTTAATATGCG

Supplementary Table 4. Primer sequences used to genotype transgenic strains.

REFERENCES

1. Moyes AJ, Khambata RS, Villar I, Bubb KJ, Baliga RS, Lumsden NG, Xiao F, Gane PJ, Rebstock AS, Worthington RJ, Simone MI, Mota F, Rivilla F, Vallejo S, Peiro C, Sanchez Ferrer CF, Djordjevic S, Caulfield MJ, MacAllister RJ, Selwood DL, Ahluwalia A, Hobbs AJ. Endothelial C-type natriuretic peptide maintains vascular homeostasis. J Clin Invest 2014;**124**(9):4039-4051.
2. Geister KA, Brinkmeier ML, Hsieh M, Faust SM, Karolyi IJ, Perosky JE, Kozloff KM, Conti M, Camper SA. A novel loss-of-function mutation in Npr2 clarifies primary role in female reproduction and reveals a potential therapy for acromesomelic dysplasia, Maroteaux type. Hum Mol Genet 2013;**22**(2):345-57.
3. Matsukawa N, Grzesik WJ, Takahashi N, Pandey KN, Pang S, Yamauchi M, Smithies O. The natriuretic peptide clearance receptor locally modulates the physiological effects of the natriuretic peptide system. Proc Natl Acad Sci U S A 1999;**96**(13):7403-7408.
4. Baliga RS, Scotton CJ, Trinder SL, Chambers RC, MacAllister RJ, Hobbs AJ. Intrinsic defence capacity and therapeutic potential of natriuretic peptides in pulmonary hypertension associated with lung fibrosis. Br J Pharmacol 2014;**171**(14):3463-3475.
5. Chun TH, Itoh H, Ogawa Y, Tamura N, Takaya K, Igaki T, Yamashita J, Doi K, Inoue M, Masatsugu K, Korenaga R, Ando J, Nakao K. Shear stress augments expression of C-type natriuretic peptide and adrenomedullin. Hypertension 1997;**29**(6):1296-302.
6. Ruetten H, Dimmeler S, Gehring D, Ihling C, Zeiher AM. Concentric left ventricular remodeling in endothelial nitric oxide synthase knockout mice by chronic pressure overload. Cardiovasc Res 2005;**66**(3):444-453.

Supplementary Figure 1

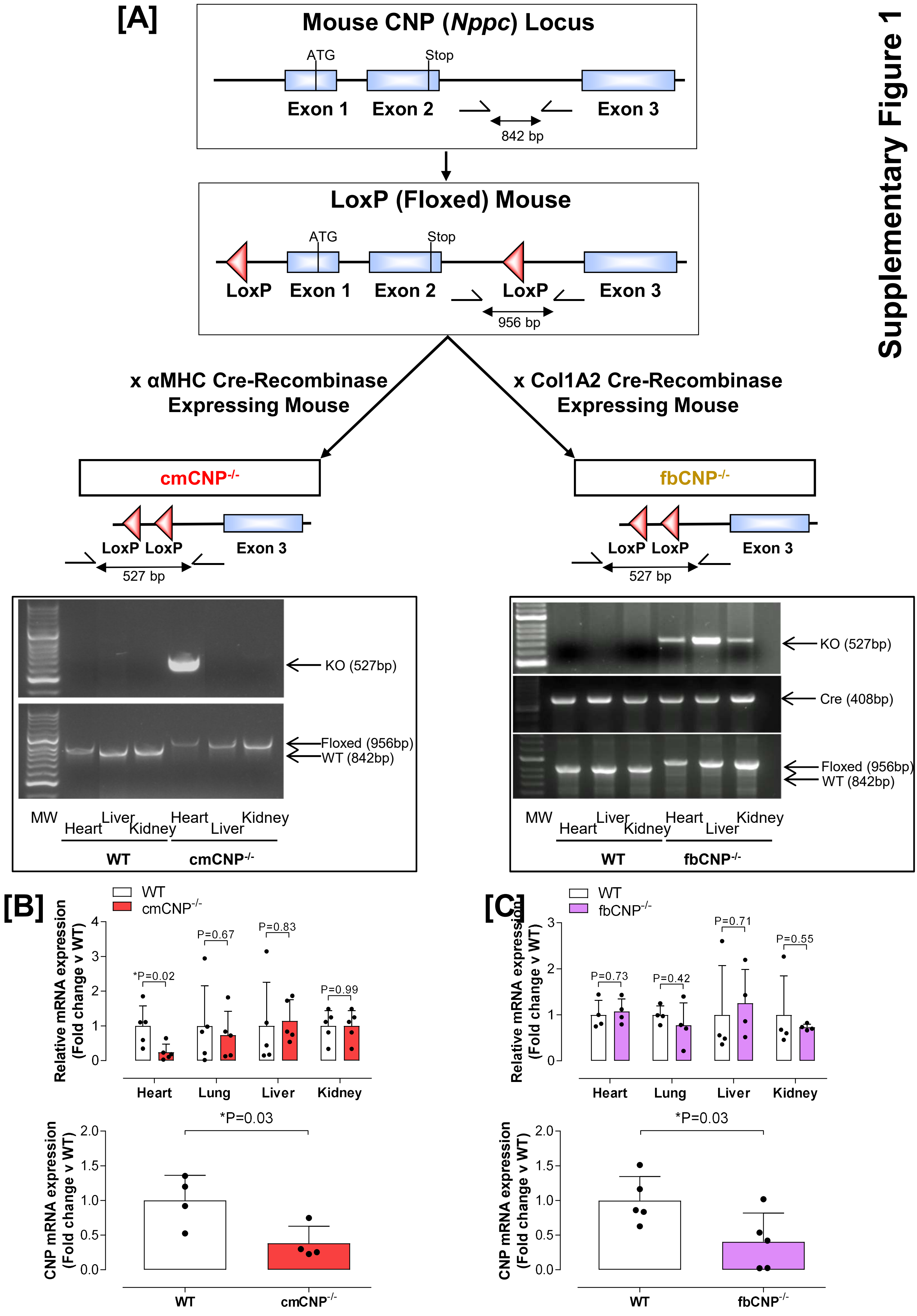

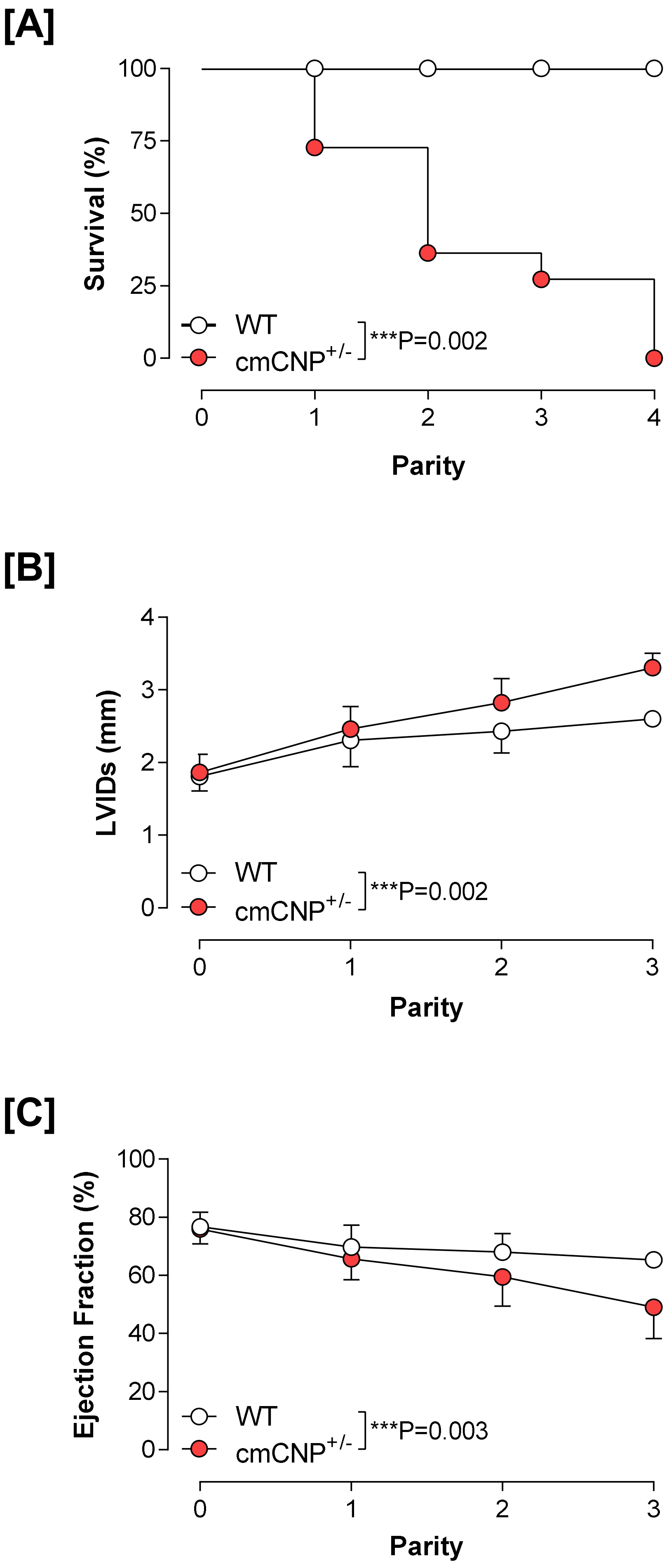

Supplementary Figure 2

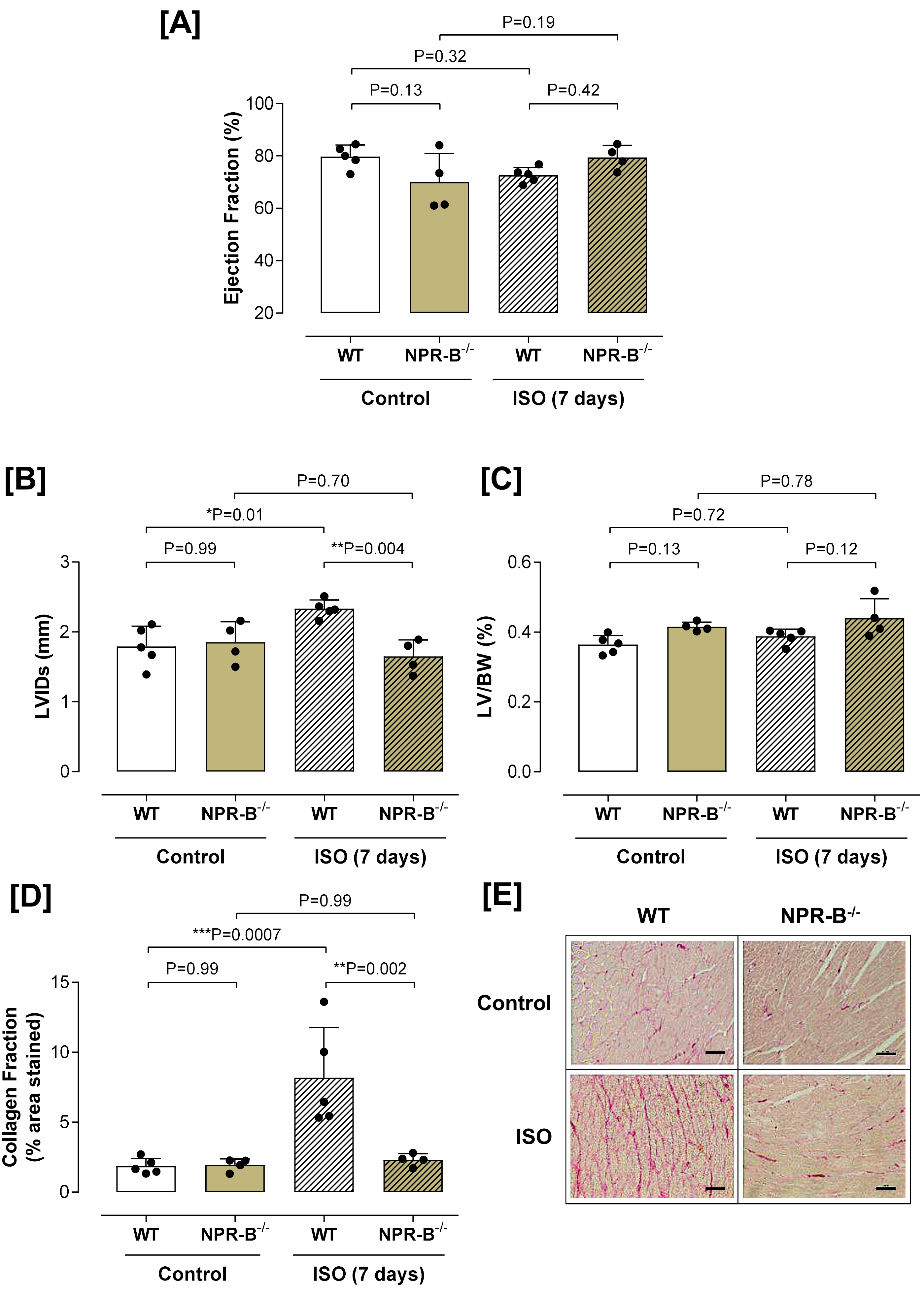

Supplementary Figure 3

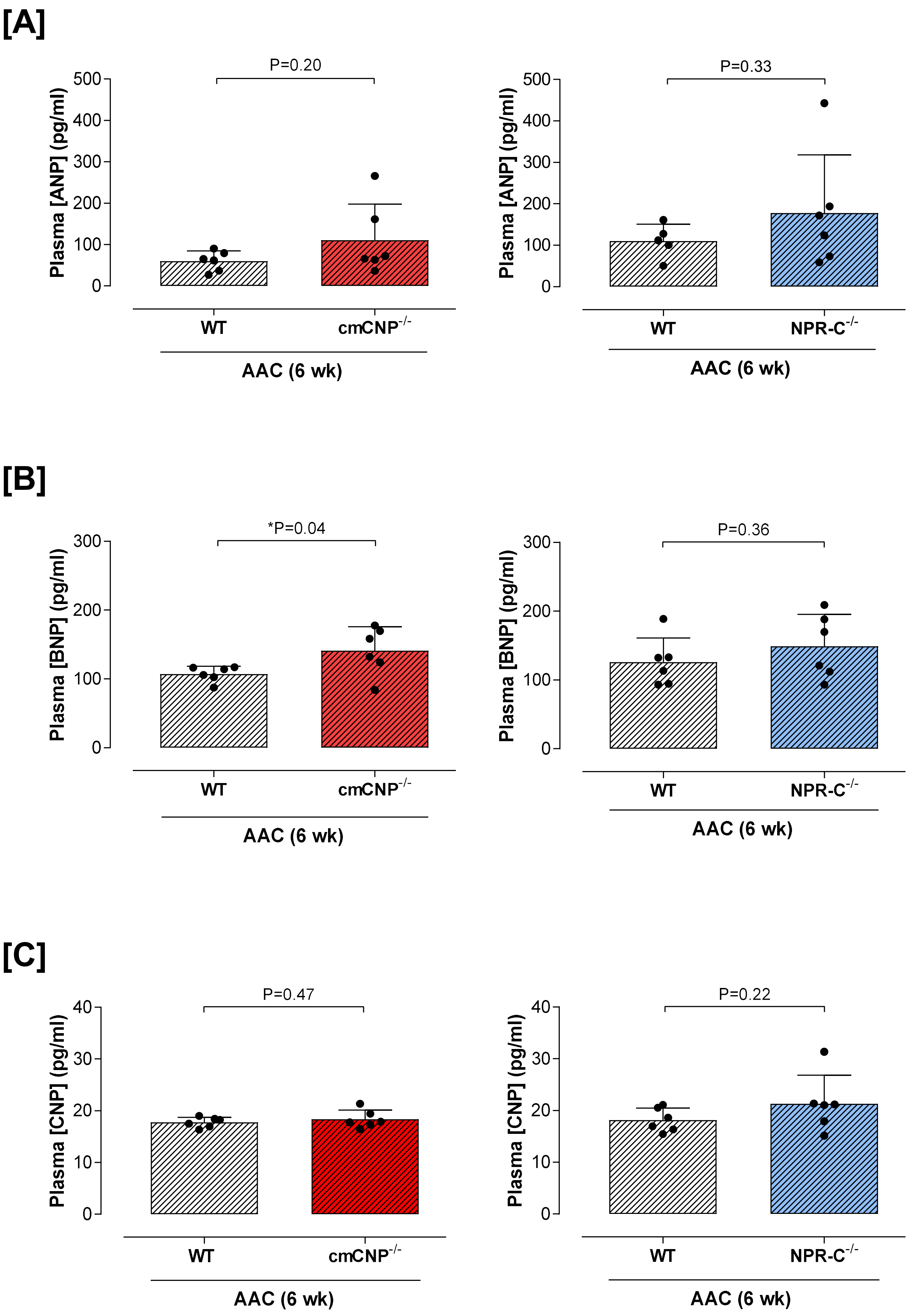

Supplementary Figure 4

Supplementary Figure 5

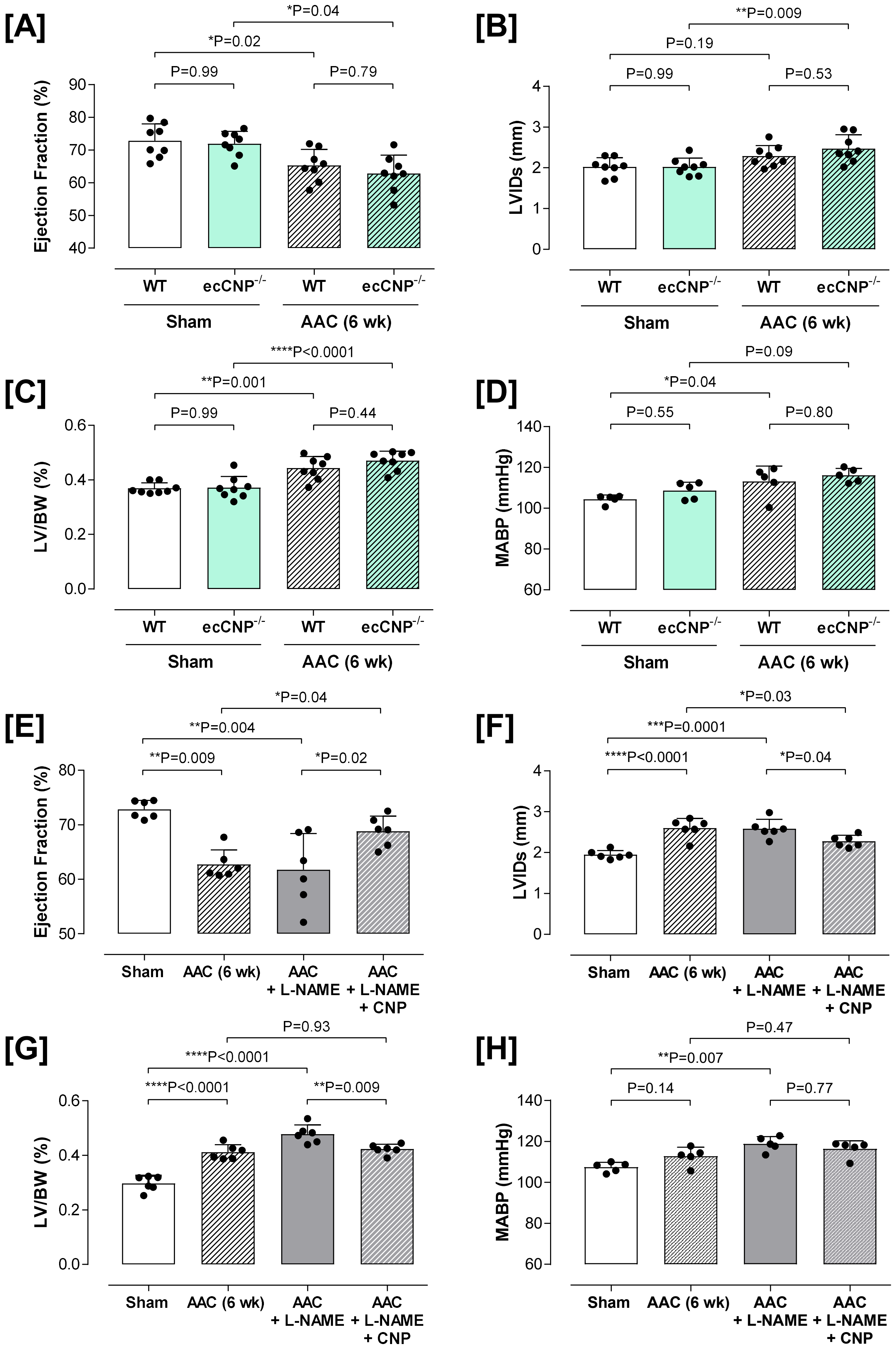

Supplementary Figure 6

[A]

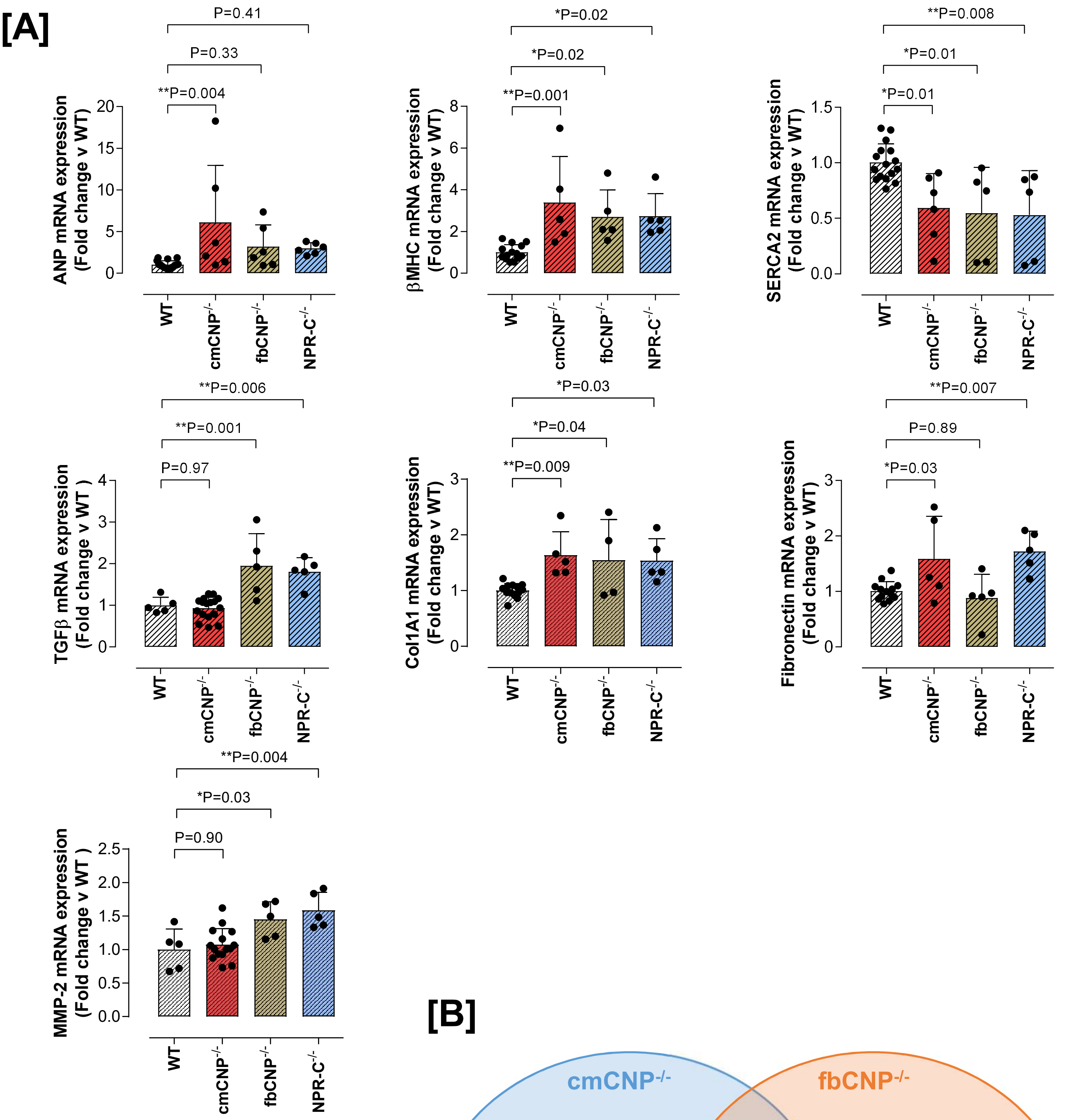

[B]

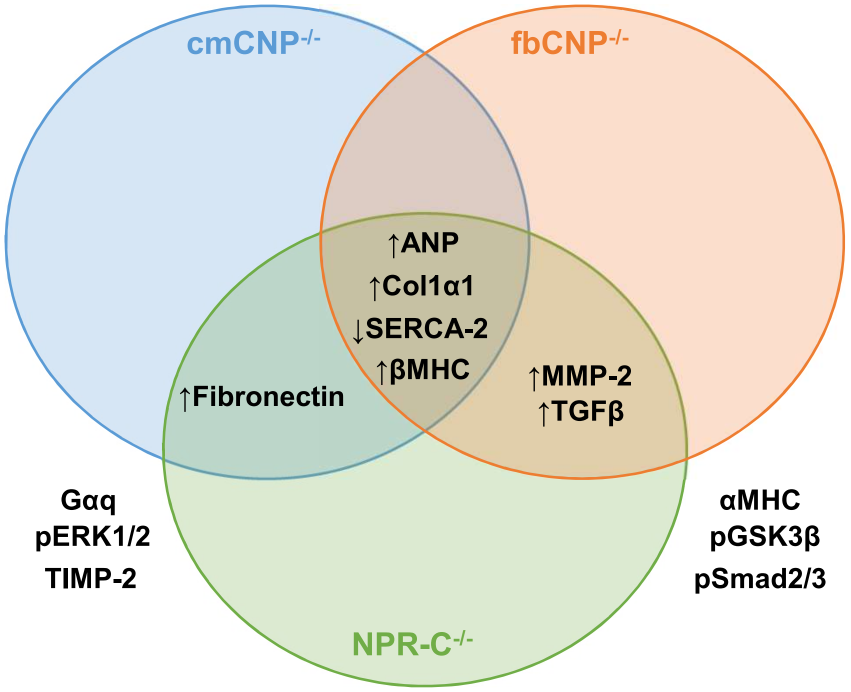

Supplementary Figure 7

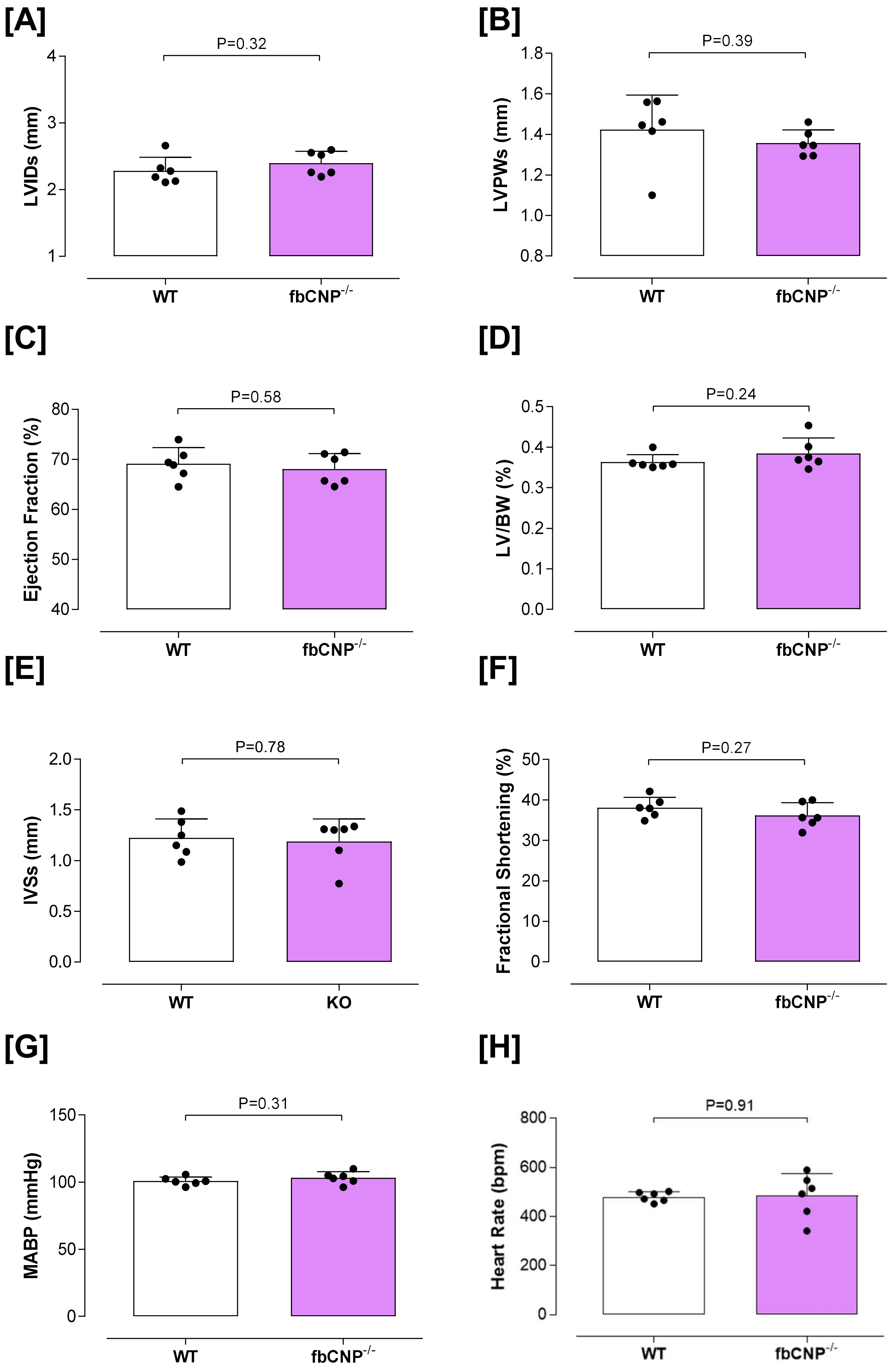

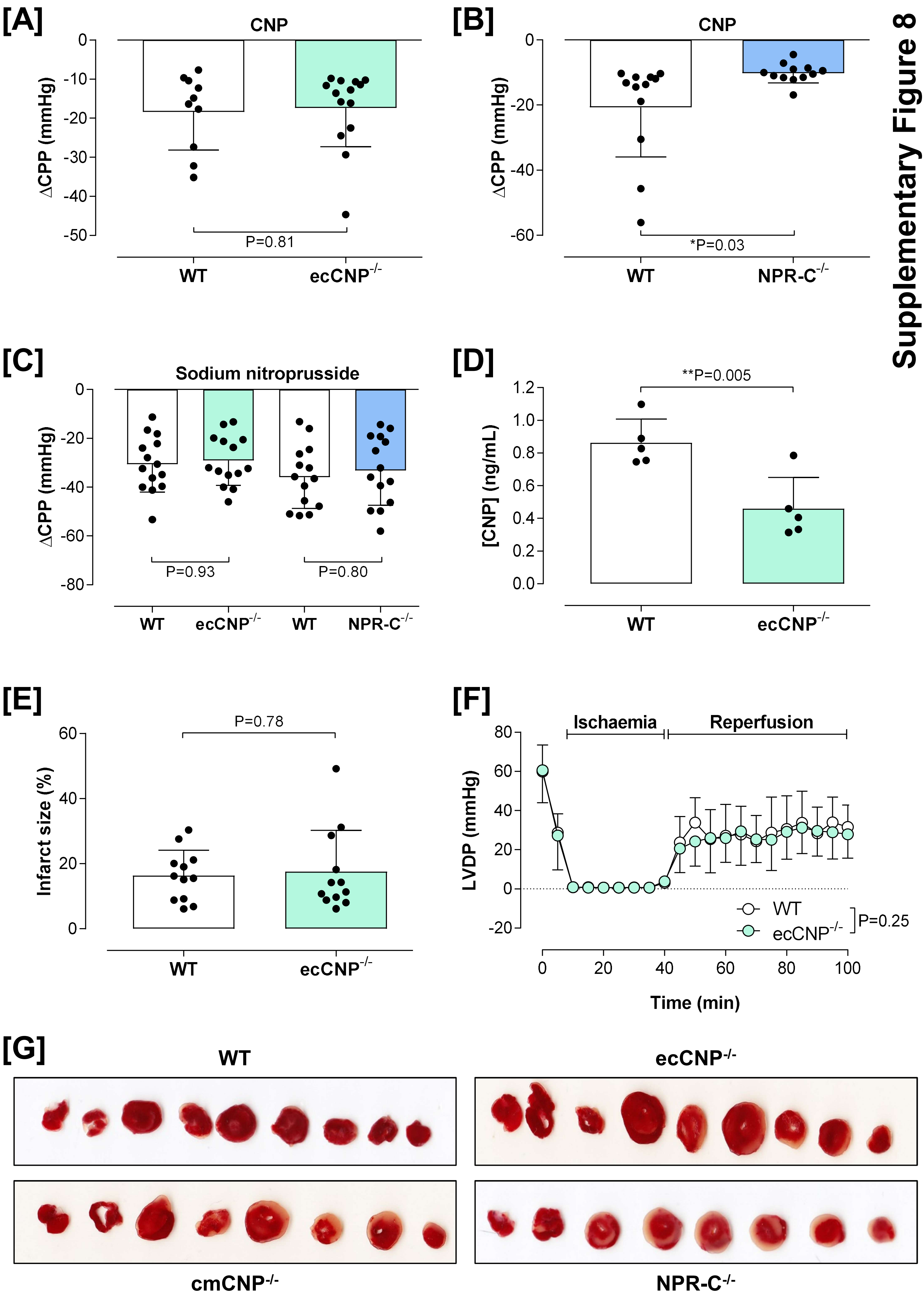

Supplementary Figure 8
